# Supplementary material for: Analysis of sub-kilobase chromatin topology reveals nano-scale regulatory interactions with variable dependence on cohesin and CTCF
Source: Nat Commun. 2022 Apr 19;13:2139. doi: 10.1038/s41467-022-29696-5 (PMC9019034; doi:10.1038/s41467-022-29696-5)
Supplement: Supplementary file 1 — Supplementary information [file 41467_2022_29696_MOESM1_ESM.pdf]

SUPPLEMENTARY INFORMATION FOR:

Analysis of sub-kilobase chromatin topology reveals nano-scale regulatory interactions with variable dependence on cohesin and CTCF

Abrar Aljahani, Peng Hua, Magdalena A. Karpinska, Kimberly Quililan, James O.J. Davies, A. Marieke Oudelaar

CONTENTS

**I.**	Supplementary Note 1	**p. 2**
**II.**	Supplementary Figures	**p. 4**
**III.**	Supplementary Tables	**p. 33**
**IV.**	References	**p. 35**

Supplementary Note 1. Comparison of the Tiled-MCC, Micro-C and Tiled-C approaches.

When comparing the Tiled-MCC and Micro-C approaches[1,2], Micro-C has the obvious advantage of generating genome-wide data, whereas Tiled-MCC has the advantage that it can generate deep data for regions of interest and supports multiplexing of samples. Therefore, in cases in which researchers are interested in characterizing 3D genome architecture in selected genomic regions in multiple cell types or genetic models, Tiled-MCC allows for deeper data generation at substantially lower sequencing costs (Figure 1, Supplementary Tables 1,2).

In addition, the Tiled-MCC and Micro-C library preparation protocols have important differences, as summarized in the diagram below.

	Micro-C	Tiled-MCC
Scale	Genome-wide	1-10 Mb
Cell preparation	Nuclear preparation	Intact cells (digitonin)
Junction enrichment	A/C (not T/G)	None
Sequencing preparation	Gel extraction di-nucleosomal band	Sonication (200 bp)
Sequencing	50-100 bp paired-end	150 bp paired-end
Junction sequencing	No	Yes
Max resolution	~200 bp	~1 bp
Sensitivity (junctions / bp)	0.8-2.2	5.3-9.3
Library complexity	Low	High

The Tiled-MCC procedure uses digitonin instead of traditional detergents for cell permeabilization and does not involve specific enrichment of ligation junctions, whereas the Micro-C procedure involves dATP and dCTP biotin labelling of digested ends and gel size selection to enrich for valid ligation events.

The use of digitonin in Tiled-MCC has the advantage that cellular and nuclear architecture are maintained and therefore increases the signal-to-noise ratio[3]. Furthermore, the loss of material in the Tiled-MCC approach is minimized by omitting biotinylation and gel size selection, which results in high library complexity. This is particularly important for targeted 3C approaches, since 3C libraries contain a limited amount of unique ligation junctions that can be sampled in regions of interest. As a result, the percentage of PCR duplicates in the enriched Tiled-MCC wild-type libraries is on average ~45%. A disadvantage of the Tiled-MCC procedure is that it does not involve specific selection of ligation junctions. As a result, not all sequenced reads are informative. Tiled-MCC libraries therefore require relatively deep sequencing considering the small proportion of the genome that is enriched (Supplementary Table 1). However, the reported statistics include sub-optimal libraries generated during the optimization of the procedure. We estimate that with our current protocol, 100-200 million reads per enriched Mb per pooled sample are sufficient for the generation of contact matrices at 500 bp resolution.

Since the Micro-C procedure involves enrichment of valid ligation junctions, a higher proportion of Micro-C reads are informative compared to Tiled-C. However, a disadvantage of the biotinylation and size selection procedures in the Micro-C approach, is that these steps are relatively inefficient and therefore substantially decrease library complexity. The Micro-C data in mES cells used for comparison in our study are derived from 38 individual samples, of which the average duplication rate in samples sequenced deeper than 100 M reads is ~40%[1]. This indicates that these libraries could provide maximum ~2-fold deeper data for regions of interest upon enrichment.

The Tiled-C approach[4] enriches for ligation junctions, since the oligonucleotides are targeted to restriction enzyme cut sites. In addition, Tiled-C libraries are digested with restriction enzymes, which generate sticky overhangs that can be ligated with very high efficiency. Therefore, the vast majority of Tiled-C reads contain useful ligation junctions. Because the complexity of Tiled-C libraries is also very high, this approach supports deep data generation at very low sequencing costs (Supplementary Table 1). However, since the resolution of Tiled-C is limited by the cut site distribution of restriction enzymes, it does not support data generation beyond a resolution of ~2 kb (Supplementary Figure 2).

In contrast to Tiled-C, both Tiled-MCC and Micro-C use MNase digestion and therefore have the potential to generate data at much higher resolution. A key difference between the Tiled-MCC and Micro-C approaches is that the Tiled-MCC procedure involves sonication of the MNase-digested 3C libraries to ~200 bp fragments and sequencing with 150 bp paired-end reads, and therefore allows for reconstruction of the precise positions of the ligation junctions. The resolution of Tiled-MCC is therefore predominantly limited by sequencing depth. Our current dataset supports analysis of localized structures at 20 bp resolution. The MCC approach, which enriches for narrow viewpoints instead of large regions as in Tiled-MCC, supports base-pair resolution analysis, because the ratio of sequencing reads per enriched bp is higher[3].

In contrast to the Tiled-MCC approach, in the Micro-C procedure, di-nucleosomal fragments are purified by gel extraction and sequenced with 50-100 bp paired-end reads. The position of the ligation junctions in Micro-C is therefore inferred, which limits the resolution to ~200 bp. Due to the size of the di-nucleosomal fragments, it is unlikely that increasing the sequencing cycles to 300 (as used in Tiled-MCC) would enable reliable identification of ligation junctions in Micro-C.

Taken together, the combination of preservation of nuclear architecture, generation of MNase-digested libraries with high complexity, deep sequencing of regions of interest, and direct identification of ligation junctions, gives Tiled-MCC important advantages compared to existing methods and enables characterization of local chromatin architecture at great depth and very high resolution (Figures 1, 2).

Supplementary Figure 1. Tiled-MCC generates highly reproducible contact matrices.

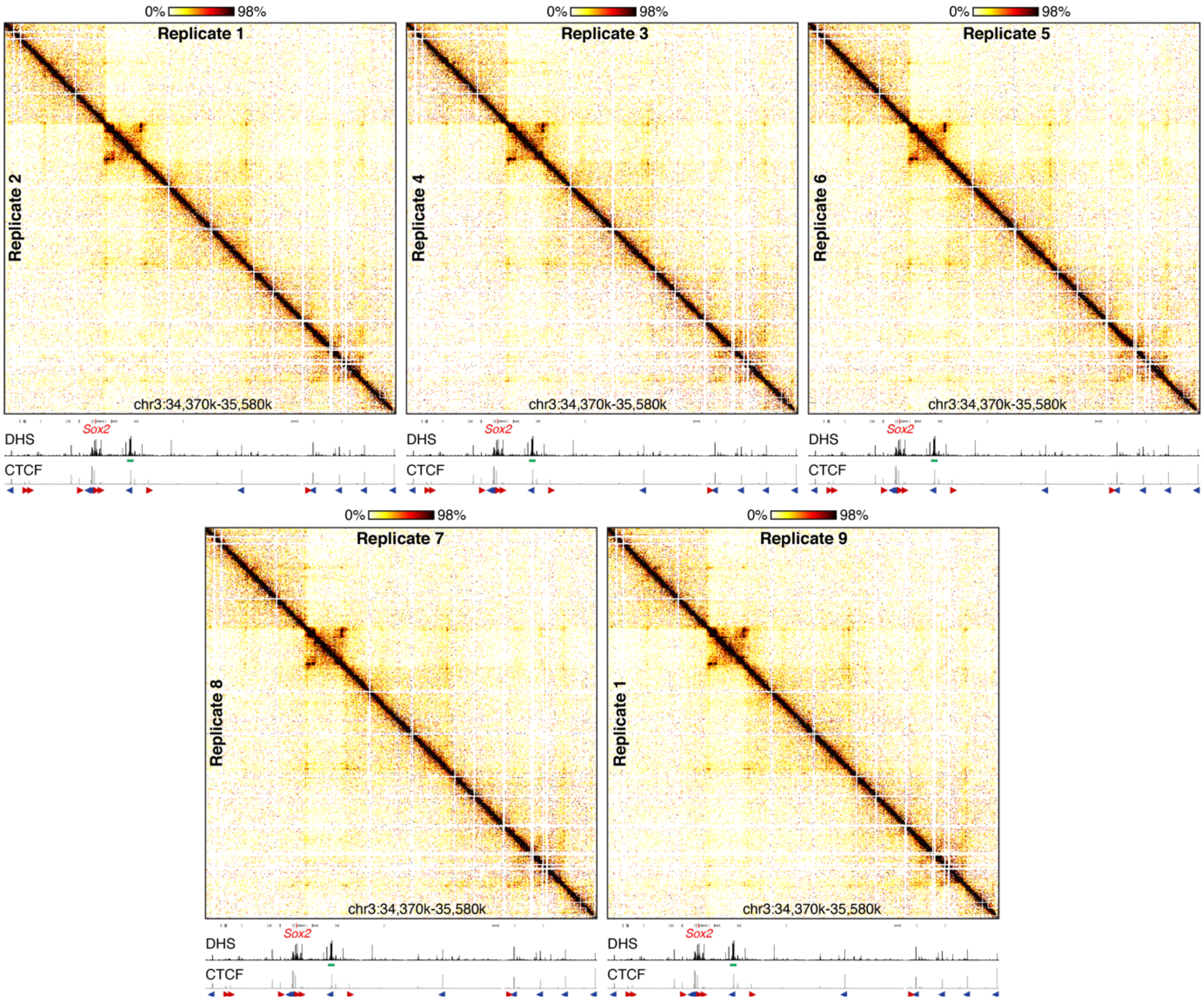

Comparison of Tiled-MCC contact matrices of the *Sox2* locus at 2 kb resolution in 9 independent replicates of wildtype mES cells. Gene annotation (*Sox2* in red, coding genes in black, non-coding genes in grey), DNase hypersensitive sites (DHS) and ChIP-seq data for CTCF are shown below the matrices. The axes of the DHS and ChIP-seq profiles are scaled to signal and have the following ranges: DHS = 0-4.46; CTCF = 0-1833. Enhancers of interest are indicated in green below the DHS profiles. The orientations of CTCF motifs at prominent CTCF-binding sites are indicated by arrowheads (forward orientation in red; reverse orientation in blue).

Supplementary Figure 2. Comparison of Tiled-MCC and Tiled-C contact matrices.

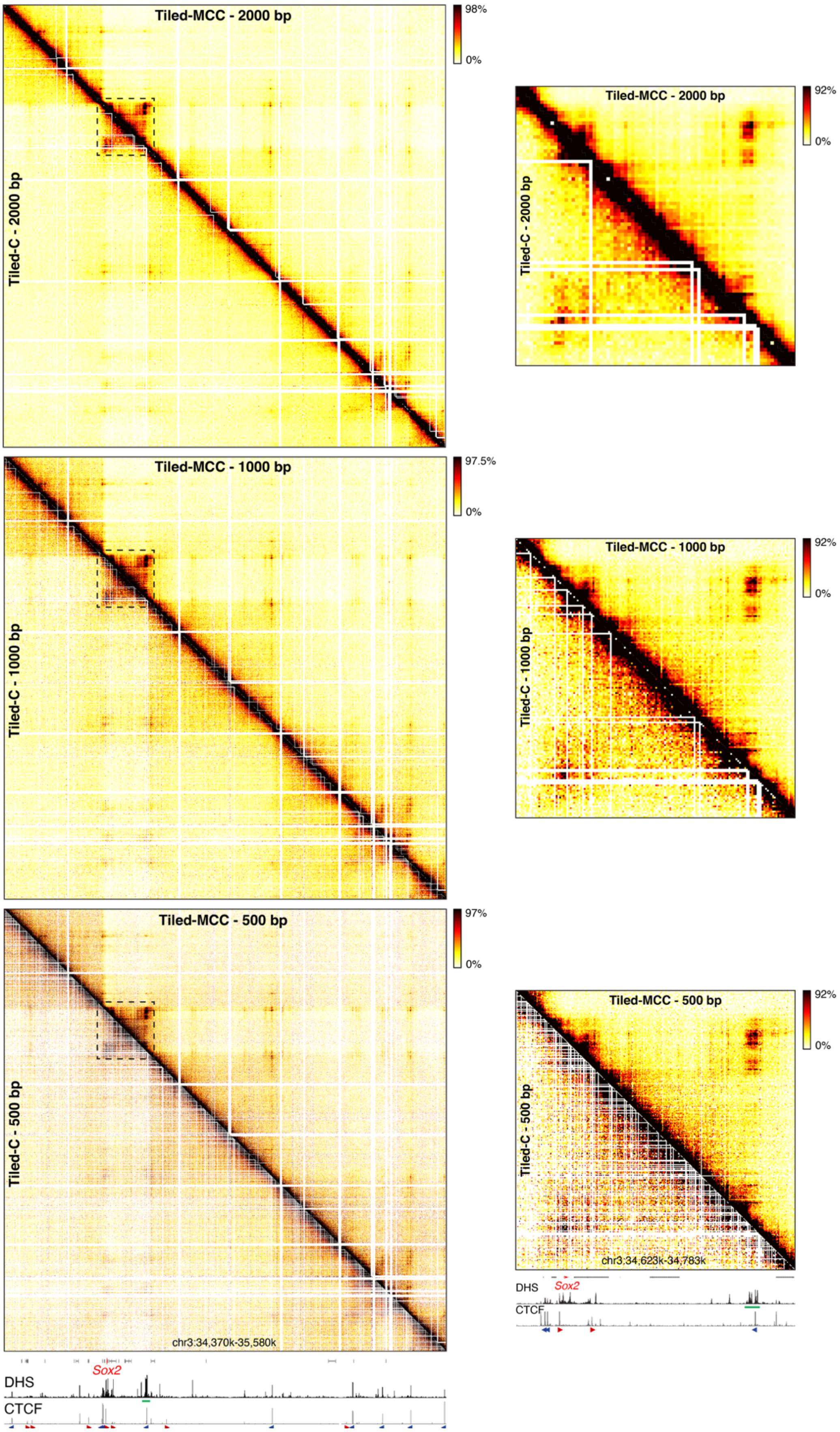

Comparison of Tiled-MCC to Tiled-C, which is the most recent and efficient approach that uses a strategy based on tiled capture oligonucleotides to enrich regions of interest in 3C libraries digested with restriction enzymes (DpnII)[4]. From top to bottom, the panels show Tiled-MCC (top-right) and Tiled-C (bottom-left) contact matrices of the *Sox2* locus at 2 kb, 1 kb and 500 bp resolution. The right panels display a zoomed view of the dashed region in the left panels to highlight the interactions between the *Sox2* promoter and its super-enhancer. Gene annotation (*Sox2* in red, coding genes in black, non-coding genes in grey), DNase hypersensitive sites (DHS) and ChIP-seq data for CTCF are shown below the matrices. The axes of the DHS and ChIP-seq profiles have the following ranges: DHS = 0-4.46; CTCF, left panel = 0-1833; CTCF, right panel = 0-300. Enhancers of interest are indicated in green below the DHS profiles. The orientations of CTCF motifs at prominent CTCF-binding sites are indicated by arrowheads (forward orientation in red; reverse orientation in blue).

Supplementary Figure 3. Comparison of Tiled-MCC and Tiled-C contact matrices at equal sequencing depth.

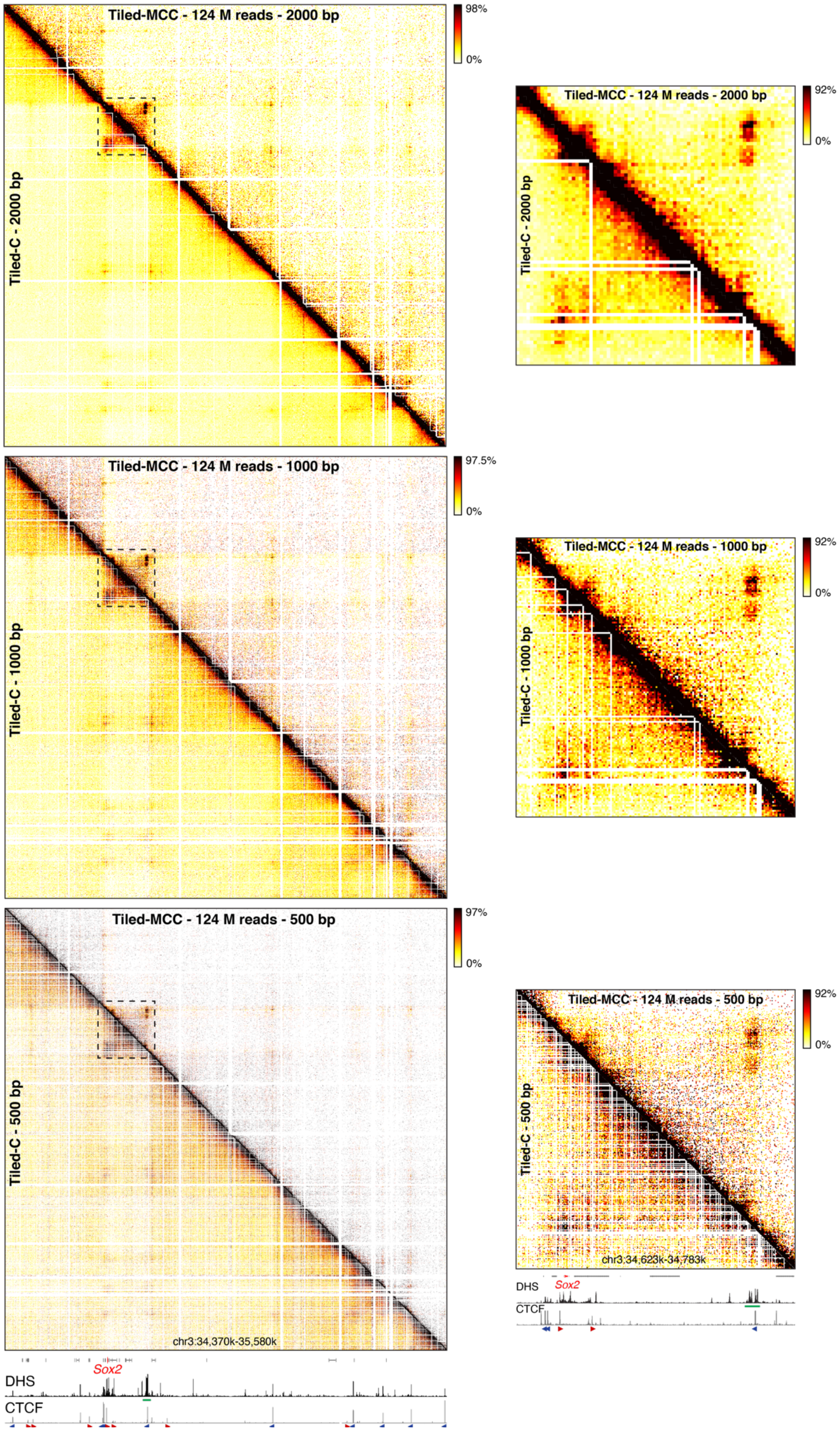

Comparison of Tiled-C contact matrices to Tiled-MCC contact matrices generated from data which is down-sampled to equal sequencing depth as the Tiled-C data (~124 million reads). From top to bottom, the panels show Tiled-MCC (top-right) and Tiled-C (bottom-left) contact matrices of the *Sox2* locus at 2 kb, 1 kb and 500 bp resolution. The right panels display a zoomed view of the dashed region in the left panels to highlight the interactions between the *Sox2* promoter and its super-enhancer. Gene annotation (*Sox2* in red, coding genes in black, non-coding genes in grey), DNase hypersensitive sites (DHS) and ChIP-seq data for CTCF are shown below the matrices. The axes of the DHS and ChIP-seq profiles have the following ranges: DHS = 0-4.46; CTCF, left panel = 0-1833; CTCF, right panel = 0-300. Enhancers of interest are indicated in green below the DHS profiles. The orientations of CTCF motifs at prominent CTCF-binding sites are indicated by arrowheads (forward orientation in red; reverse orientation in blue).

Supplementary Figure 4. Analysis of Tiled-MCC contact matrices at a sequencing depth of 500 million reads.

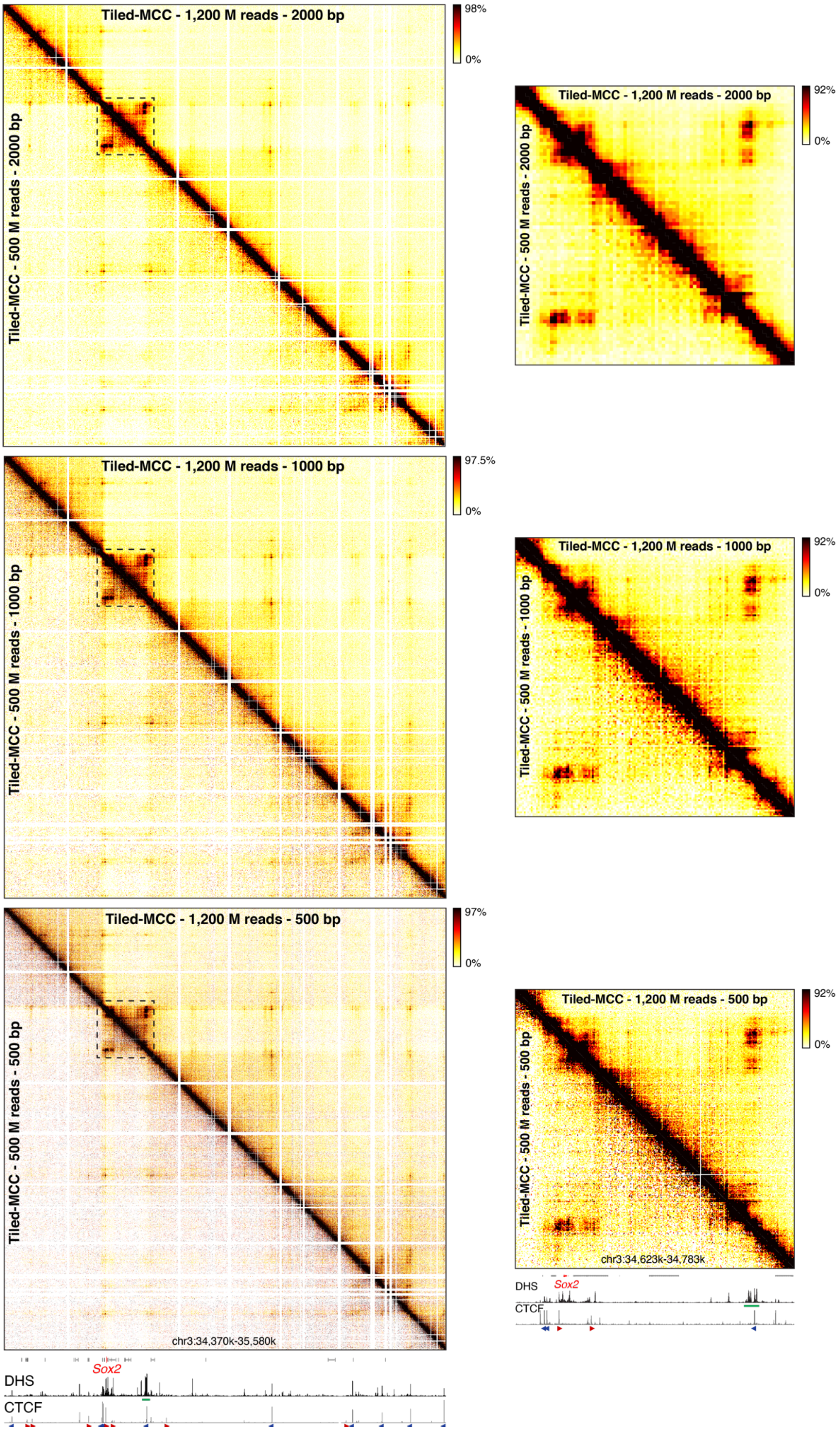

Comparison of Tiled-MCC contact matrices generated from all data (~1,200 million reads; top-right) and data down-sampled to 500 million reads (bottom-left). From top to bottom, the panels show Tiled-MCC contact matrices of the *Sox2* locus at 2 kb, 1 kb and 500 bp resolution. The right panels display a zoomed view of the dashed region in the left panels to highlight the interactions between the *Sox2* promoter and its super-enhancer. Gene annotation (*Sox2* in red, coding genes in black, non-coding genes in grey), DNase hypersensitive sites (DHS) and ChIP-seq data for CTCF are shown below the matrices. The axes of the DHS and ChIP-seq profiles have the following ranges: DHS = 0-4.46; CTCF, left panel = 0-1833; CTCF, right panel = 0-300. Enhancers of interest are indicated in green below the DHS profiles. The orientations of CTCF motifs at prominent CTCF-binding sites are indicated by arrowheads (forward orientation in red; reverse orientation in blue).

Supplementary Figure 5. Analysis of Tiled-MCC contact matrices at a sequencing depth of 250 million reads.

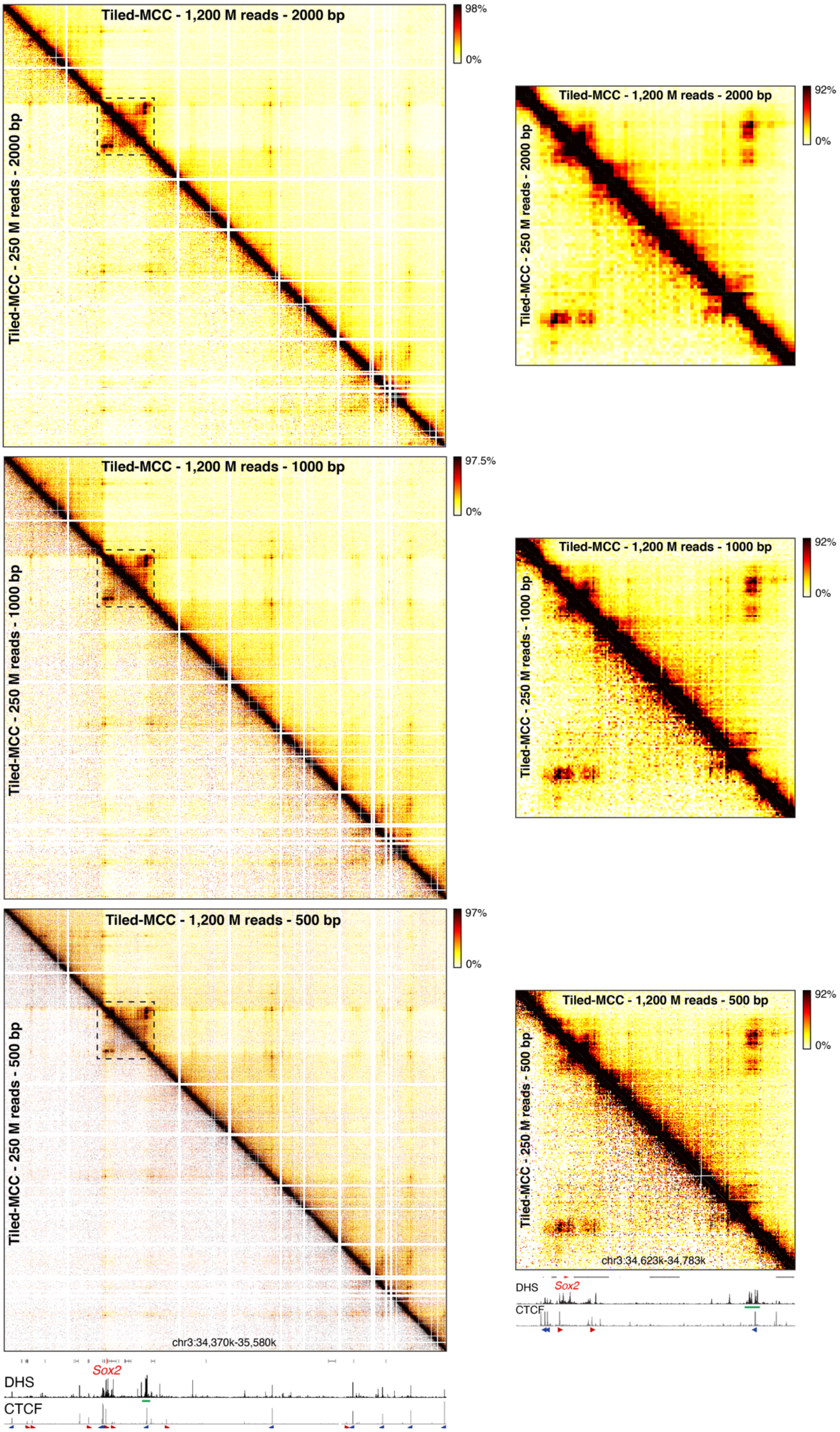

Comparison of Tiled-MCC contact matrices generated from all data (~1,200 million reads; top-right) and data down-sampled to 250 million reads (bottom-left). From top to bottom, the panels show Tiled-MCC contact matrices of the *Sox2* locus at 2 kb, 1 kb and 500 bp resolution. The right panels display a zoomed view of the dashed region in the left panels to highlight the interactions between the *Sox2* promoter and its super-enhancer. Gene annotation (*Sox2* in red, coding genes in black, non-coding genes in grey), DNase hypersensitive sites (DHS) and ChIP-seq data for CTCF are shown below the matrices. The axes of the DHS and ChIP-seq profiles have the following ranges: DHS = 0-4.46; CTCF, left panel = 0-1833; CTCF, right panel = 0-300. Enhancers of interest are indicated in green below the DHS profiles. The orientations of CTCF motifs at prominent CTCF-binding sites are indicated by arrowheads (forward orientation in red; reverse orientation in blue).

Supplementary Figure 6. Comparison of high-resolution ICE-normalized contact matrices and density plots of Tiled-MCC data.

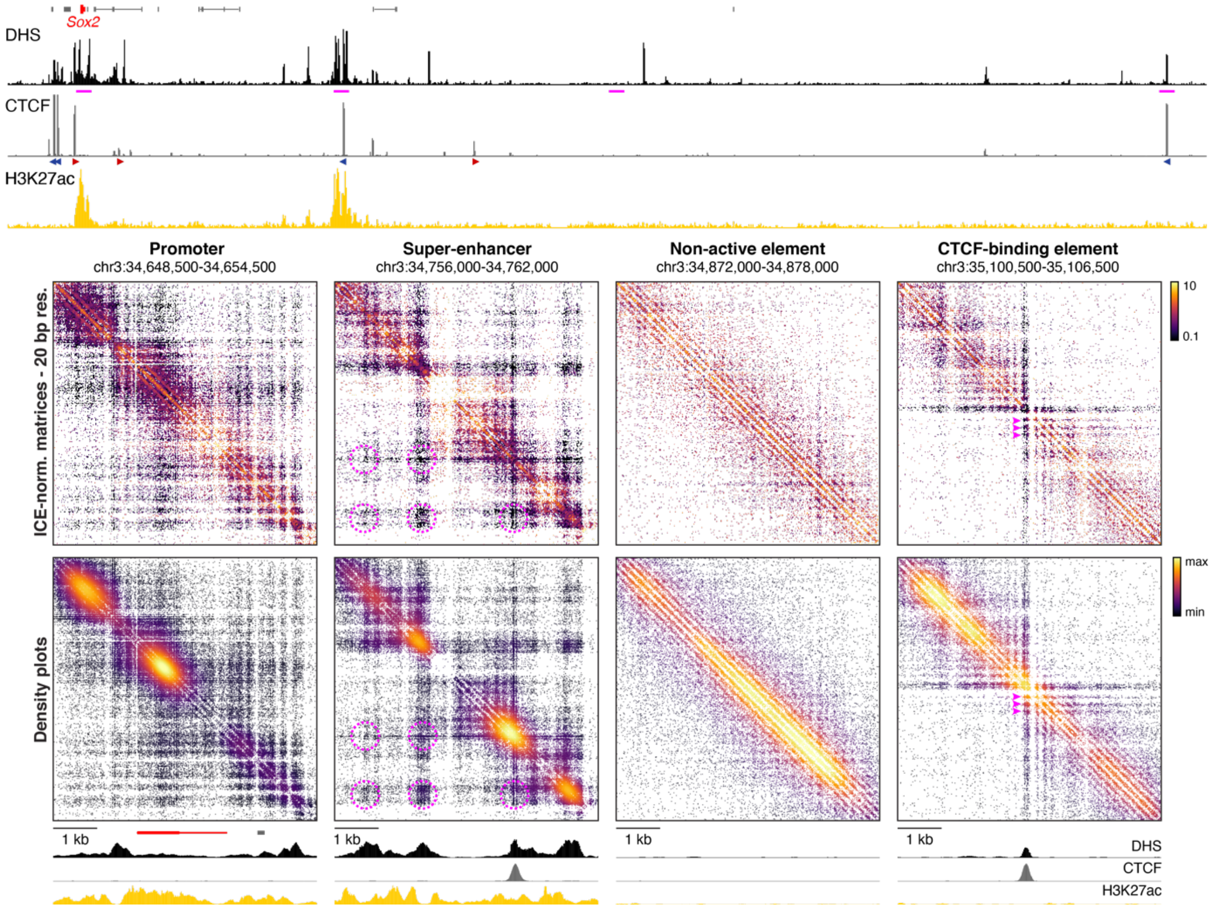

Comparison of high-resolution (20 bp) ICE-normalized contact matrices (top) and density plots (bottom) displaying Tiled-MCC ligation junctions in the *Sox2* locus. Gene annotation (*Sox2* in red, coding genes in black, non-coding genes in grey), DNase hypersensitive sites (DHS), and ChIP-seq data for CTCF and H3K27ac for the extended *Sox2* locus are shown above the matrices. The 6 kb regions covered in the contact matrices are highlighted with magenta bars below the top DHS profile, and show a gene promoter, super-enhancer, non-active element and CTCF-binding element. The axes of the top and bottom DHS and ChIP-seq profiles for CTCF and H3K27ac are fixed and have the following ranges: DHS = 0-5; CTCF = 0-1500; H3K27ac = 0-50. The orientations of CTCF motifs at prominent CTCF-binding sites are indicated in the top panel by arrowheads (forward orientation in red; reverse orientation in blue). The magenta highlights in the contact matrix and density plot covering the super-enhancer indicate enriched interactions between DHSs; the magenta highlights in the contact matrix and density plot covering the CTCF-binding element indicate phased nucleosomes.

The density plots allow for direct visualization of the precise locations of the ligation junctions, but are not straightforward to interpret quantitatively and normalize. This leads to an unequal signal at the diagonal, which likely reflects variability in MNase cutting density and capture efficiency across regions. Visualization of the data in very high-resolution ICE-normalized contact matrices allows for correction of unequal coverage. These matrices show the same patterns of chromatin interactions away from the diagonal as compared to the density plots, but without artifacts due to unequal coverage at the diagonal.

Supplementary Figure 7. Tiled-MCC micro-topology analysis at different sequencing depths.

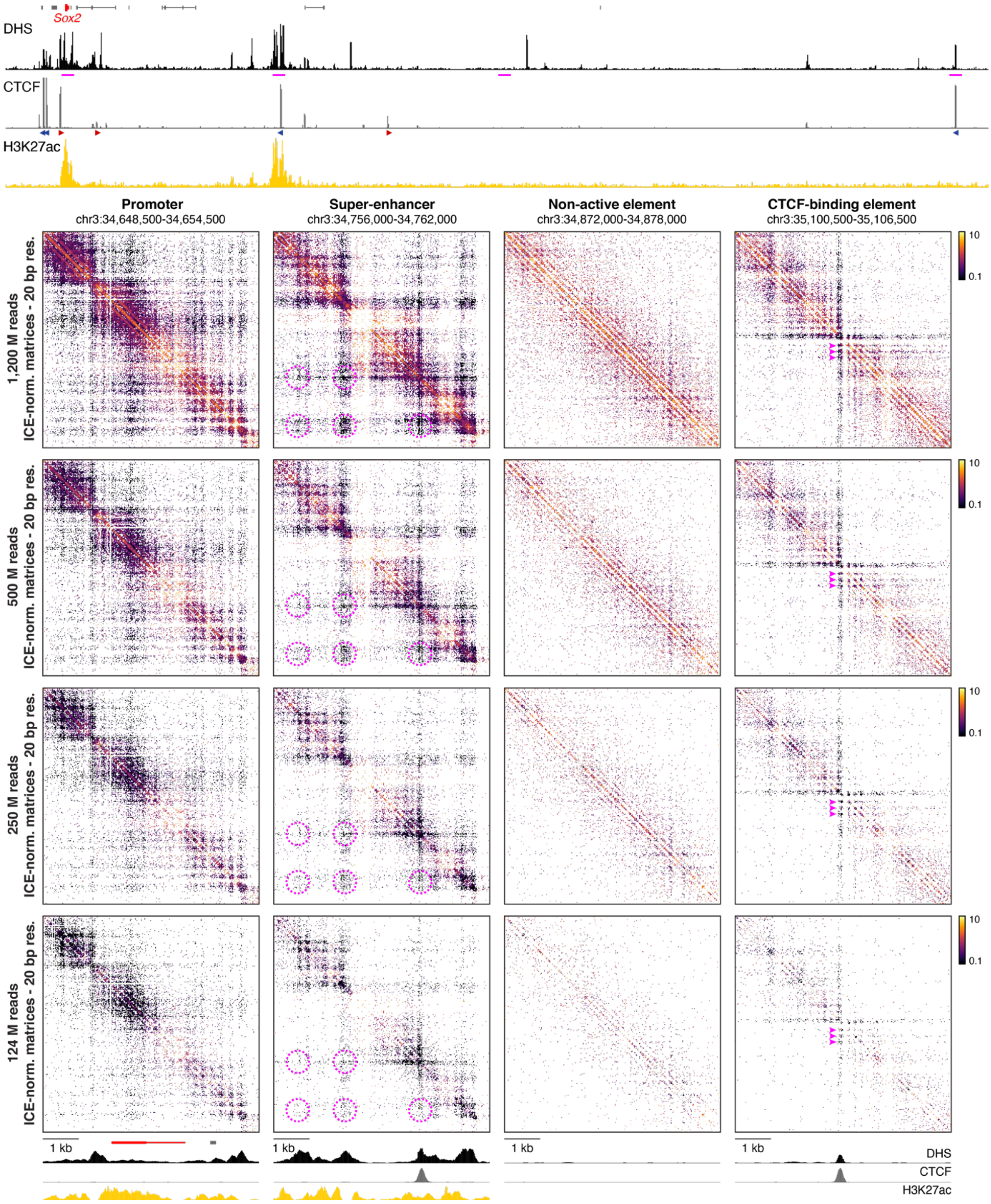

Comparison of high-resolution (20 bp) contact matrices of the *Sox2* locus generated from the complete Tiled-MCC dataset (~1,200 million reads) and data down-sampled to 500 million reads, 250 million reads, and 124 million reads. Gene annotation (*Sox2* in red, coding genes in black, non-coding genes in grey), DNase hypersensitive sites (DHS), and ChIP-seq data for CTCF and H3K27ac for the extended *Sox2* locus are shown above the matrices. The 6 kb regions covered in the contact matrices are highlighted with magenta bars below the top DHS profile, and show a gene promoter, super-enhancer, non-active element and CTCF-binding element. The axes of the top and bottom DHS and ChIP-seq profiles for CTCF and H3K27ac are fixed and have the following ranges: DHS = 0-5; CTCF = 0-1500; H3K27ac = 0-50. The orientations of CTCF motifs at prominent CTCF-binding sites are indicated in the top panel by arrowheads (forward orientation in red; reverse orientation in blue). The magenta highlights in the contact matrices covering the super-enhancer indicate enriched interactions between DHSs; the magenta highlights in the contact matrices covering the CTCF-binding element indicate phased nucleosomes.

The fine-scale features of the micro-topologies are clearly visible at a depth of 500 million and 250 million reads, which is equivalent to 100-200 million reads per enriched Mb. At a depth of 124 million reads (equivalent to the Tiled-C data depth), the features are more difficult to distinguish, though still visible.

Supplementary Figure 8. Comparison of Tiled-MCC and Tiled-C micro-topology analysis.

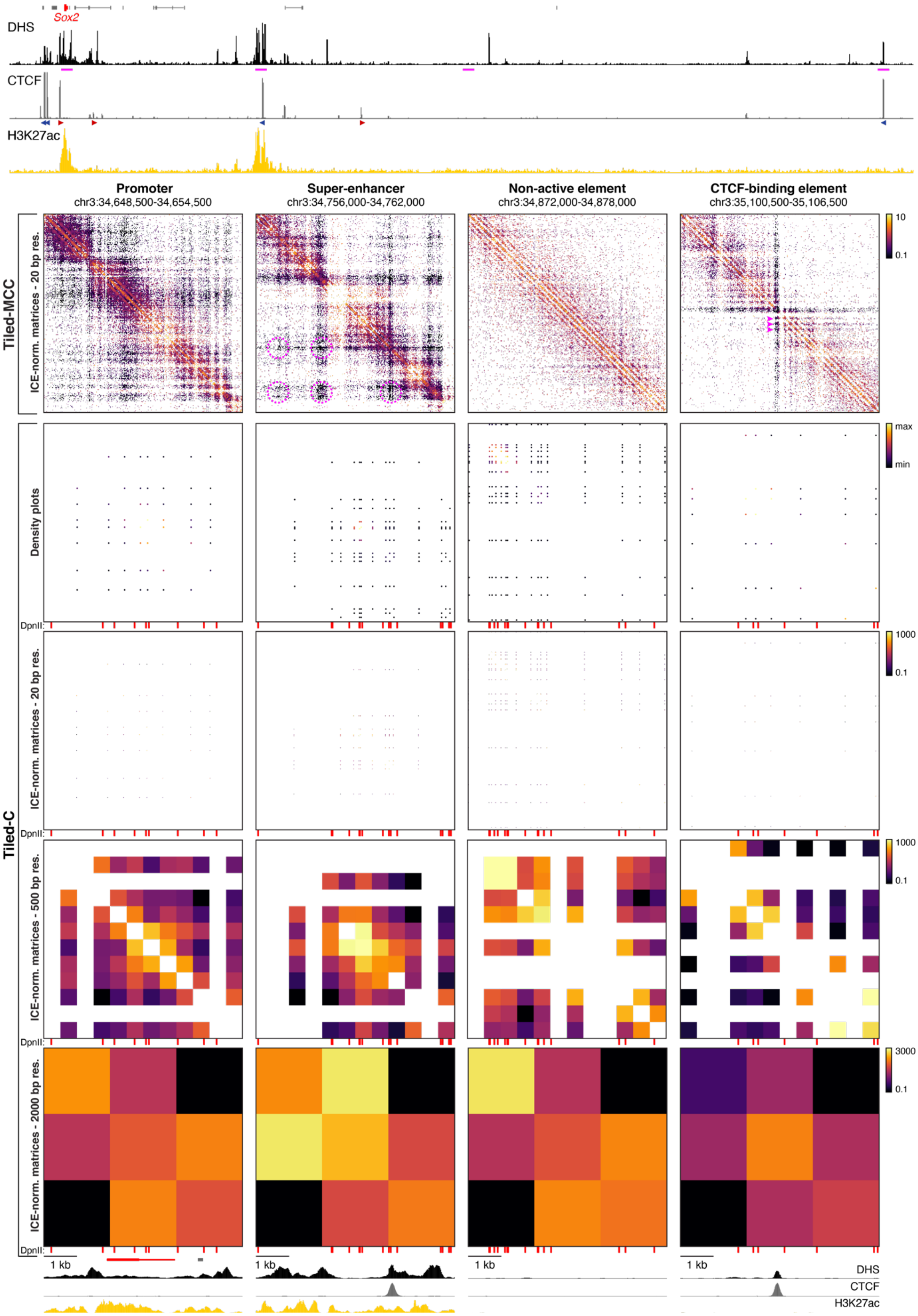

The contact matrices show ICE-normalized ligation junctions identified by Tiled-MCC in the *Sox2* locus at 20 bp resolution at the top and ligation junctions identified by Tiled-C at the bottom. The Tiled-C data are visualized as density plots and ICE-normalized contact matrices at 20 bp, 500 bp, and 2000 bp resolution, with the distribution of the DpnII restriction sites indicated with red vertical bars directly underneath the plots and matrices. Gene annotation (*Sox2* in red, coding genes in black, non-coding genes in grey), DNase hypersensitive sites (DHS), and ChIP-seq data for CTCF and H3K27ac for the extended *Sox2* locus are shown above the matrices. The 6 kb regions covered in the contact matrices are highlighted with magenta bars below the top DHS profile, and show a gene promoter, super-enhancer, non-active element and CTCF-binding element. The axes of the top and bottom DHS and ChIP-seq profiles for CTCF and H3K27ac are fixed and have the following ranges: DHS = 0-5; CTCF = 0-1500; H3K27ac = 0-50. The orientations of CTCF motifs at prominent CTCF-binding sites are indicated in the top panel by arrowheads (forward orientation in red; reverse orientation in blue). The magenta highlights in the Tiled-MCC contact matrix covering the super-enhancer indicate enriched interactions between DHSs; the magenta highlights in the Tiled-MCC contact matrix covering the CTCF-binding element indicate phased nucleosomes.

Since Tiled-C ligation junctions can only be formed at the edges of restriction fragments, the density plots show patterns representing the distribution of restriction sites across the genome. Note that the data are plotted to the middle of the restriction fragments for these analyses. Analysis at 20 bp resolution shows similar patterns, because only bins located at the intersections between restriction sites contain data; the majority of the bins are empty, as Tiled-C does not support analysis at very high resolution due to the resolution barrier imposed by the restriction fragment distribution across the genome. Analysis at 500 bp resolution is also difficult to interpret, as many bins remain empty due to the limited resolution of Tiled-C. At a resolution of 2000 bp, all bins in the selected regions contain data, but the resolution is too low for the analysis of fine-scale features of chromatin organization, irrespective of sequencing depth.

Supplementary Figure 9. Comparison of Tiled-MCC and Micro-C micro-topology analysis.

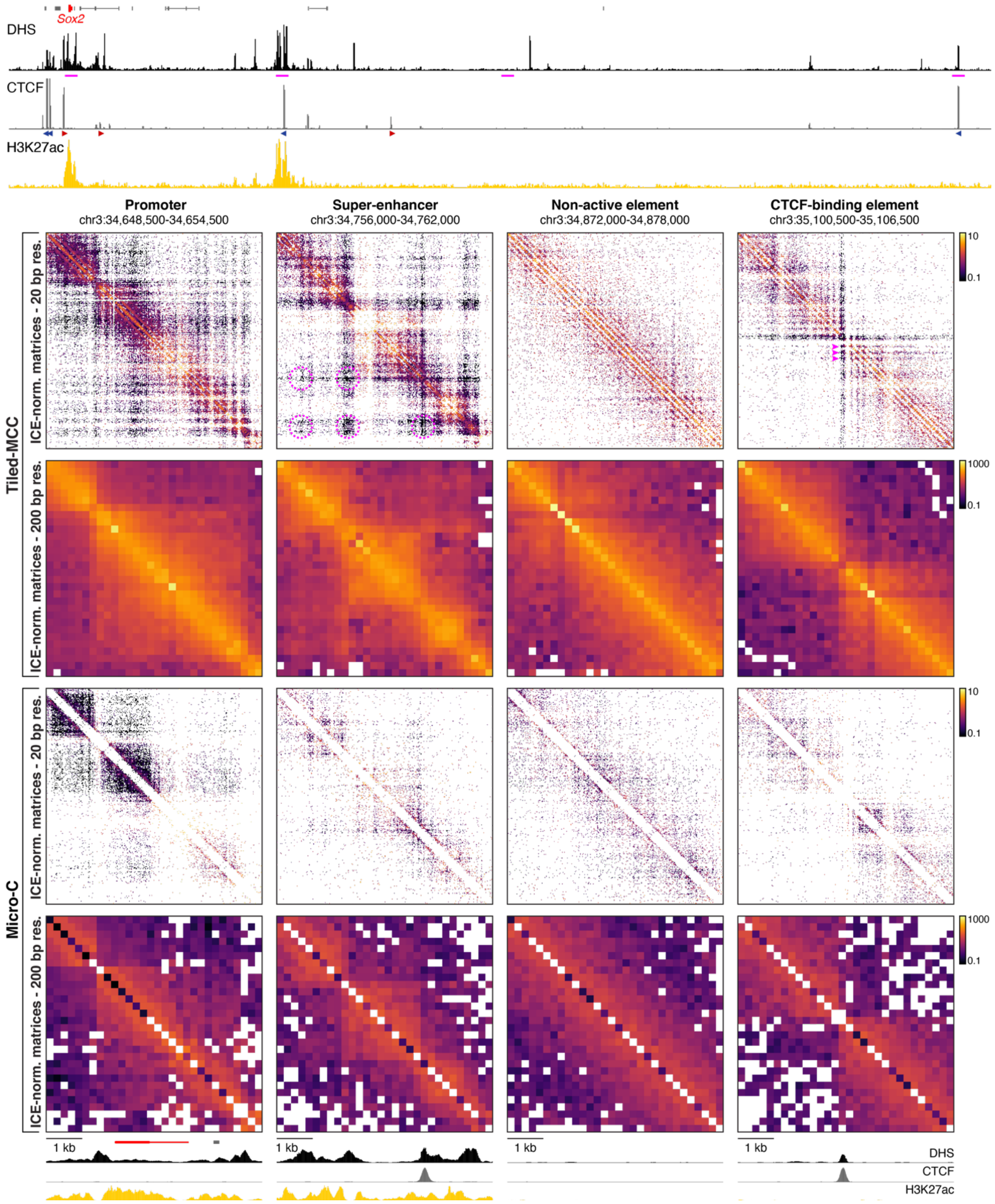

Comparison of contact matrices of the *Sox2* locus generated with Tiled-MCC (top) and Micro-C (bottom) data at 20 bp and 200 bp resolution. Gene annotation (*Sox2* in red, coding genes in black, non-coding genes in grey), DNase hypersensitive sites (DHS), and ChIP-seq data for CTCF and H3K27ac for the extended *Sox2* locus are shown above the matrices. The 6 kb regions covered in the contact matrices are highlighted with magenta bars below the top DHS profile, and show a gene promoter, super-enhancer, non-active element and CTCF-binding element. The axes of the top and bottom DHS and ChIP-seq profiles for CTCF and H3K27ac are fixed and have the following ranges: DHS = 0-5; CTCF = 0-1500; H3K27ac = 0-50. The orientations of CTCF motifs at prominent CTCF-binding sites are indicated in the top panel by arrowheads (forward orientation in red; reverse orientation in blue). The magenta highlights in the Tiled-MCC contact matrix at 20 bp resolution covering the super-enhancer indicate enriched interactions between DHSs; the magenta highlights in the Tiled-MCC contact matrix at 20 bp resolution covering the CTCF-binding element indicate phased nucleosomes.

Since the Micro-C procedure does not allow for direct identification of ligation junctions, the location of the junctions is inferred with a deviation of ~200 bp. Visualization of the Micro-C data beyond 200 bp resolution therefore does not result in biologically meaningful fine-scale interaction patterns. At 200 bp resolution, the patterns detected in the Tiled-MCC and Micro-C data are similar. However, very fine-scale features of chromatin structure cannot be appreciated at this resolution.

Supplementary Figure 10. Quality control of Tiled-MCC data in RAD21-AID mES cells.

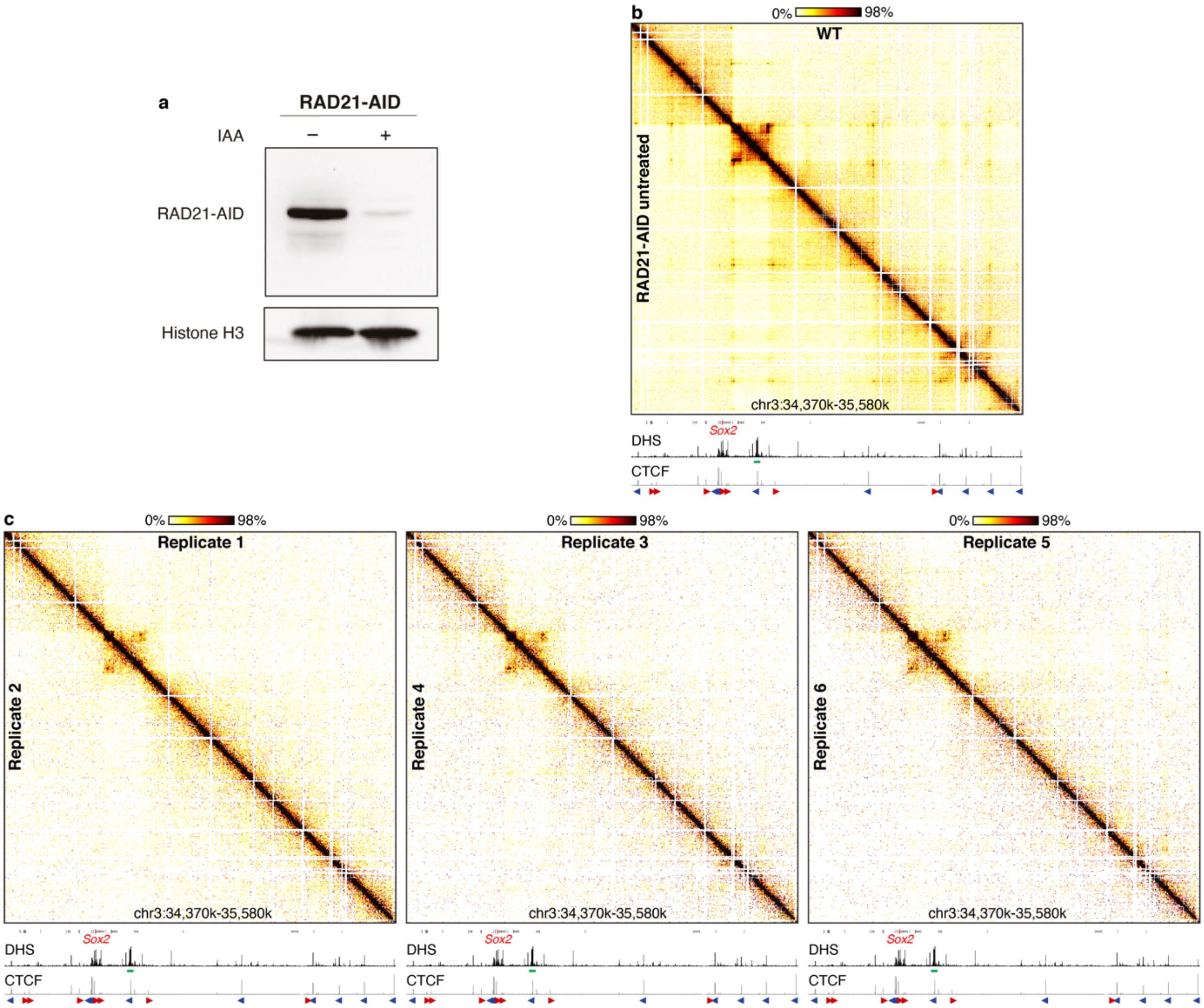

a. A representative immunoblot blot for RAD21 in the RAD21-AID mES cell line without treatment and after treatment with indole-3-acetic acid (IAA; auxin) for 6 hours. Histone H3 is shown as a loading control. Uncropped scans including molecular weight markers are provided in the Source Data files. The experiment was repeated independently three times with similar results. **b.** Comparison of Tiled-MCC contact matrices of the *Sox2* locus at 1 kb resolution in wildtype (WT) mES cells and untreated RAD21-AID mES cells. Gene annotation (*Sox2* in red, coding genes in black, non-coding genes in grey), DNase hypersensitive sites (DHS) and ChIP-seq data for CTCF are shown below the matrices. The axes of the DHS and ChIP-seq profiles are scaled to signal and have the following ranges: DHS = 0-4.46; CTCF = 0-1833. Enhancers of interest are indicated in green below the DHS profiles. The orientations of CTCF motifs at prominent CTCF-binding sites are indicated by arrowheads (forward orientation in red; reverse orientation in blue). **c.** Comparison of Tiled-MCC contact matrices of the *Sox2* locus at 2 kb resolution in 6 independent replicates of auxin-treated RAD21-AID mES cells. Annotation below the matrix is the same as in panel b.

Supplementary Figure 11. Cohesin depletion results in reduced enhancer-promoter interactions in the *Prdm14* locus.

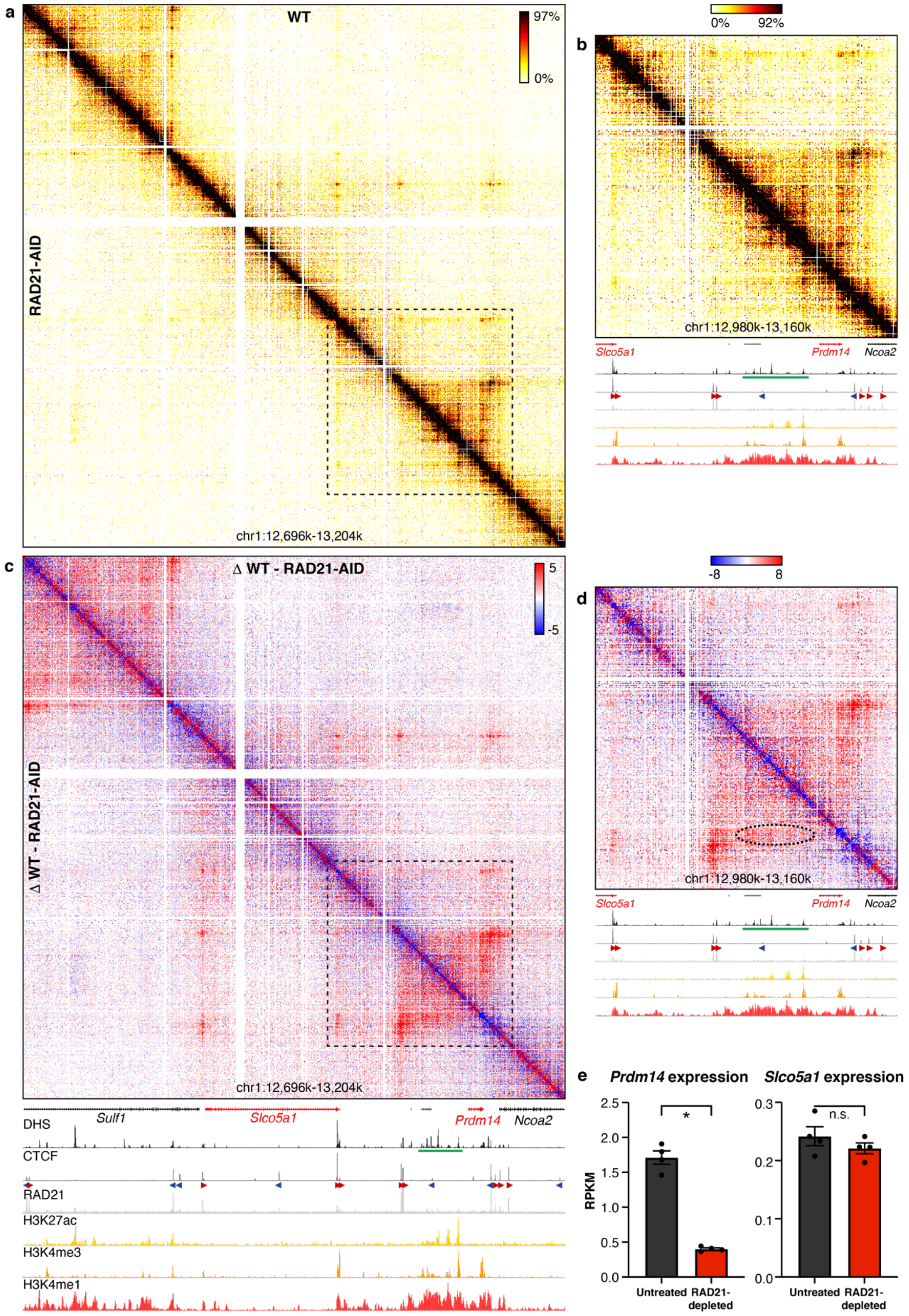

a. Tiled-MCC contact matrices of the *Prdm14* locus in wild type (WT) mES cells (top-right) and auxin-treated RAD21-AID mES cells (bottom-left) at 500 bp resolution. **b.** Zoomed view of the dashed region in panel a to highlight the interactions between the *Prdm14* promoter and the super-enhancer. **c.** Differential contact matrix of the *Prdm14* locus in which interactions enriched in WT mES cells are indicated in red and interactions enriched in auxin-treated RAD21-AID mES cells are indicated in blue. **d.** Zoomed view of the dashed region in panel c to highlight the interactions between the *Prdm14* promoter and the super-enhancer. **a-d.** Gene annotation (genes of interest in red, coding genes in black, non-coding genes in grey), DNase hypersensitive sites (DHS) and ChIP-seq data for CTCF, Cohesin (RAD21), H3K27ac, H3K4me3 and H3K4me1 are shown below the matrices. The axes of the DHS and ChIP-seq profiles below the contact matrices are scaled to signal and have the following ranges: DHS = 0-6.45; CTCF = 0-2167; RAD21 = 0-3032; H3K27ac = 0-44; H3K4me3 = 0-26; H3K4me1 = 0-2170. Enhancers of interest are indicated in green below the DHS profiles. The orientations of CTCF motifs at prominent CTCF-binding sites are indicated by arrowheads (forward orientation in red; reverse orientation in blue). The dashed oval in panel d highlights the interactions between the *Prdm14* promoter and the super-enhancer, which are reduced in the auxin-treated RAD21-AID mES cells compared to the WT mES cells. **e.** Expression of *Prdm14* and *Slc2a3* in untreated (left) and auxin-treated (right) RAD21-AID mES cells, derived from RNA-seq data, normalized for reads per kilobase of transcript, per million mapped reads (RPKM). The bars represent the average of n=4 replicates and the error bars indicate the standard error of the mean. Significant (*) and non-significant (n.s.) changes in expression are indicated. *Prdm14*: P = 3.64E-45; *Slco5a1*: P = 0.388 (calculated using DESeq2 analysis and adjusted for multiple comparisons, as previously decribed[5]). Source data are provided as a Source Data file.

Depletion of cohesin reduces the expression of *Prdm14* with ~75%. In comparison to the other gene loci which we have investigated, this is a relatively large effect. It has previously been shown that deletion of the super-enhancer reduces *Prdm14* expression by more than 95%[6] and that deletions of individual enhancer elements decrease *Prdm14* expression up to ~80% in mES cells[7,8].

Supplementary Figure 12. Cohesin depletion results in reduced enhancer-promoter interactions in the *Nanog* and *Pou5f1* loci.

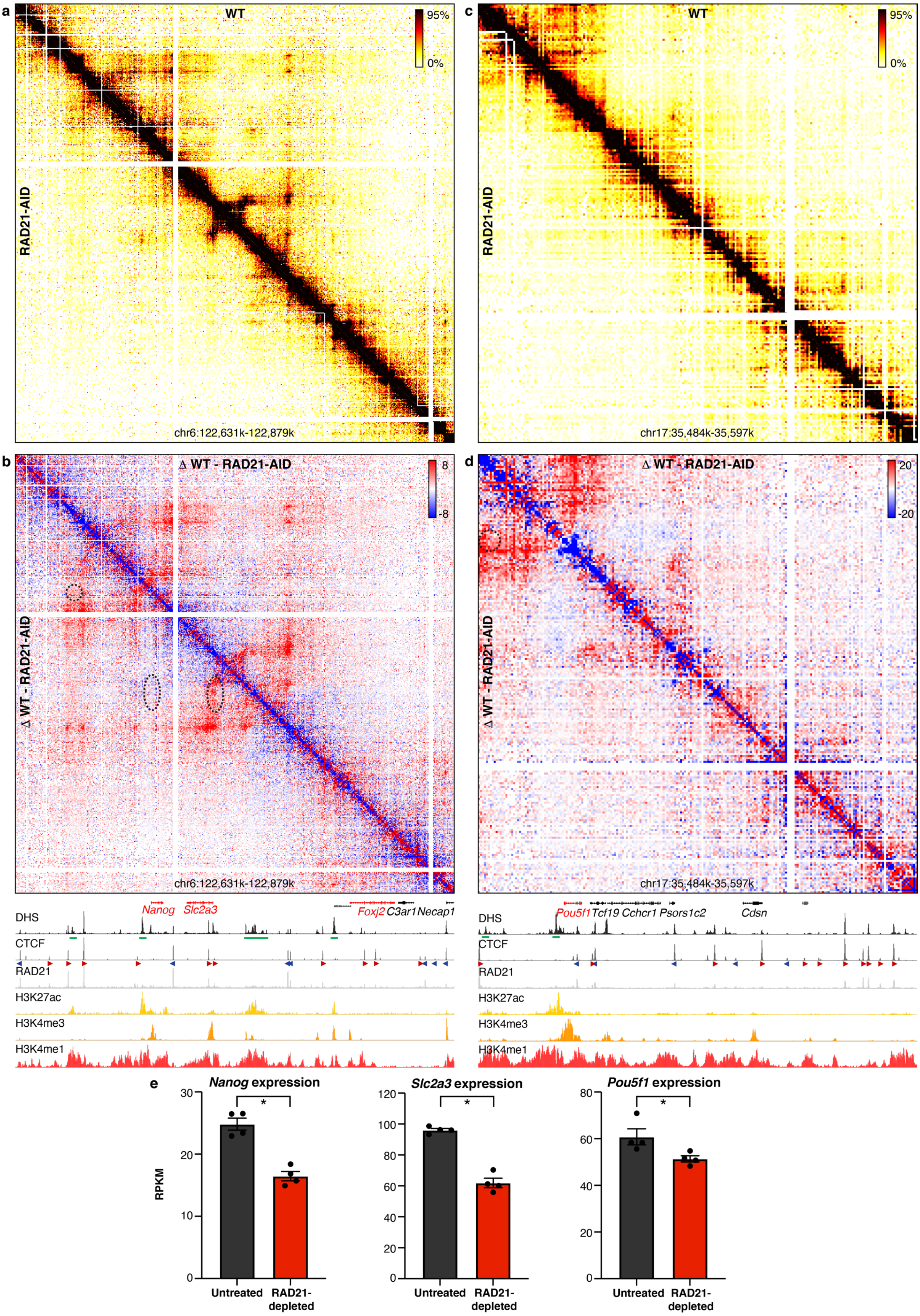

a. Tiled-MCC contact matrices of the *Nanog* locus in wild type (WT) mES cells (top-right) and auxin-treated RAD21-AID mES cells (bottom-left) at 500 bp resolution. **b.** Differential contact matrix of the *Nanog* locus in which interactions enriched in WT mES cells are indicated in red and interactions enriched in auxin-treated RAD21-AID mES cells are indicated in blue. The dashed circle and ovals highlight the following interactions of interest (from left to right): *Nanog* promoter and far upstream enhancer; *Nanog* promoter and downstream super-enhancer; *Slc2a3* promoter and downstream super-enhancer. The interactions between the *Nanog* promoter and the far upstream enhancer and between the *Slc2a3* promoter and the downstream super-enhancer are reduced upon cohesin depletion, whereas the interactions between the *Nanog* promoter and the downstream super-enhancer do not appear to be strongly impacted upon cohesin depletion. **c.** Tiled-MCC contact matrices of the *Pou5f1* locus in wild type (WT) mES cells (top-right) and auxin-treated RAD21-AID mES cells (bottom-left) at 500 bp resolution. **d.** Differential contact matrix of the *Pou5f1* locus in which interactions enriched in WT mES cells are indicated in red and interactions enriched in auxin-treated RAD21-AID mES cells are indicated in blue. The dashed circle indicates the interactions between the *Pou5f1* promoter and the far upstream enhancer, which are reduced in the auxin-treated RAD21-AID mES cells compared to the WT mES cells. **a-d.** Gene annotation (genes of interest in red, coding genes in black, non-coding genes in grey), DNase hypersensitive sites (DHS) and ChIP-seq data for CTCF, Cohesin (RAD21), H3K27ac, H3K4me3 and H3K4me1 are shown below the matrices. The axes of the DHS and ChIP-seq profiles below the contact matrices are scaled to signal and have the following ranges: DHS: *Nanog* = 0-10.25, *Pou5f1* = 0-8.65; CTCF: *Nanog* = 0-3092, *Pou5f1* = 0-2349; RAD21: *Nanog* = 0-3414, *Pou5f1* = 0-4107; H3K27ac: *Nanog* = 0-58, *Pou5f1* = 0-81; H3K4me3: *Nanog* = 0-90, *Pou5f1* = 0-64; H3K4me1: *Nanog* = 0-2064, *Pou5f1* = 0-1641. Enhancers of interest are indicated in green below the DHS profiles. The orientations of CTCF motifs at prominent CTCF-binding sites are indicated by arrowheads (forward orientation in red; reverse orientation in blue). **e.** Expression of *Nanog*, *Slc2a3* and *Pou5f1* in untreated (left) and auxin-treated (right) RAD21-AID mES cells, derived from RNA-seq data, normalized for reads per kilobase of transcript, per million mapped reads (RPKM). The bars represent the average of n=4 replicates and the error bars indicate the standard error of the mean. Significant (*) changes in expression are indicated. *Nanog*: P = 2.76E-14; *Slc2a3*: P = 3.64E-16; *Pou5f1*: P = 0.00586 (calculated using DESeq2 analysis and adjusted for multiple comparisons, as previously decribed[5]). Source data are provided as a Source Data file.

Cohesin depletion reduces *Nanog* expression by ~33%. It has previously been shown that homozygous deletion of the far upstream enhancer reduces *Nanog* expression by ~50%, whereas removal of the downstream super-enhancer does not seem to have a big impact on *Nanog* expression in mES cells[9]. Homozygous deletion of the proximal upstream enhancer appears lethal in mES cells, where heterozygous deletion of this elements reduces *Nanog* expression by about ~50%[9]. Expression of *Pou5f1* is reduced with ~15% upon cohesin depletion. There is no information available for the impact of enhancer deletions on *Pou5f1* expression in mES cells for comparison.

Supplementary Figure 13. Quality control of Tiled-MCC data in CTCF-AID mES cells.

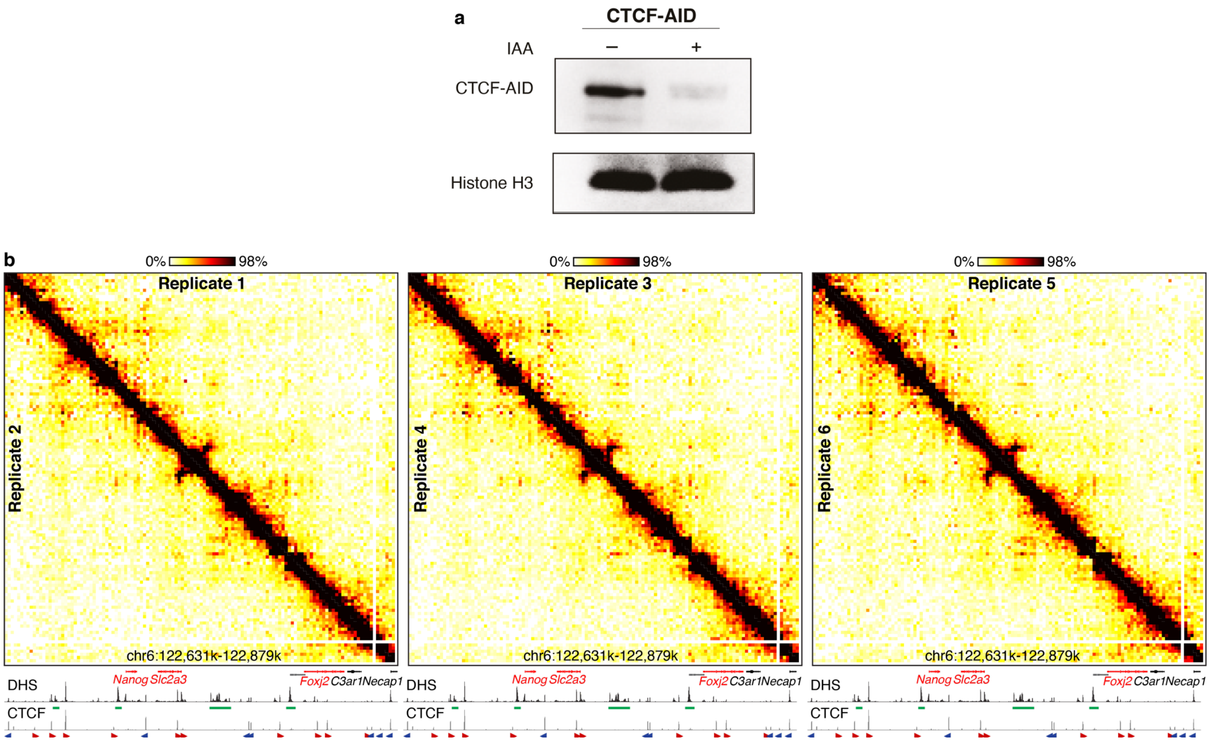

a. A representative immunoblot for CTCF in the CTCF-AID mES cell line without treatment and after treatment with indole-3-acetic acid (IAA; auxin) for 48 hours. Histone H3 is shown as a loading control. Uncropped scans including molecular weight markers are provided in the Source Data files. The experiment was repeated independently three times with similar results. **b.** Comparison of Tiled-MCC contact matrices of the *Nanog* locus at 2 kb resolution in 6 independent replicates of auxin-treated CTCF-AID mES cells. Gene annotation (genes of interest in red, coding genes in black, non-coding genes in grey), DNase hypersensitive sites (DHS) and ChIP-seq data for CTCF are shown below the matrices. The axes of the DHS and ChIP-seq profiles are scaled to signal and have the following ranges: DHS = 0-10.25; CTCF = 0-3092. Enhancers of interest are indicated in green below the DHS profiles. The orientations of CTCF motifs at prominent CTCF-binding sites are indicated by arrowheads (forward orientation in red; reverse orientation in blue).

Supplementary Figure 14. CTCF depletion results in ectopic enhancer-promoter interactions in the *Prdm14* locus.

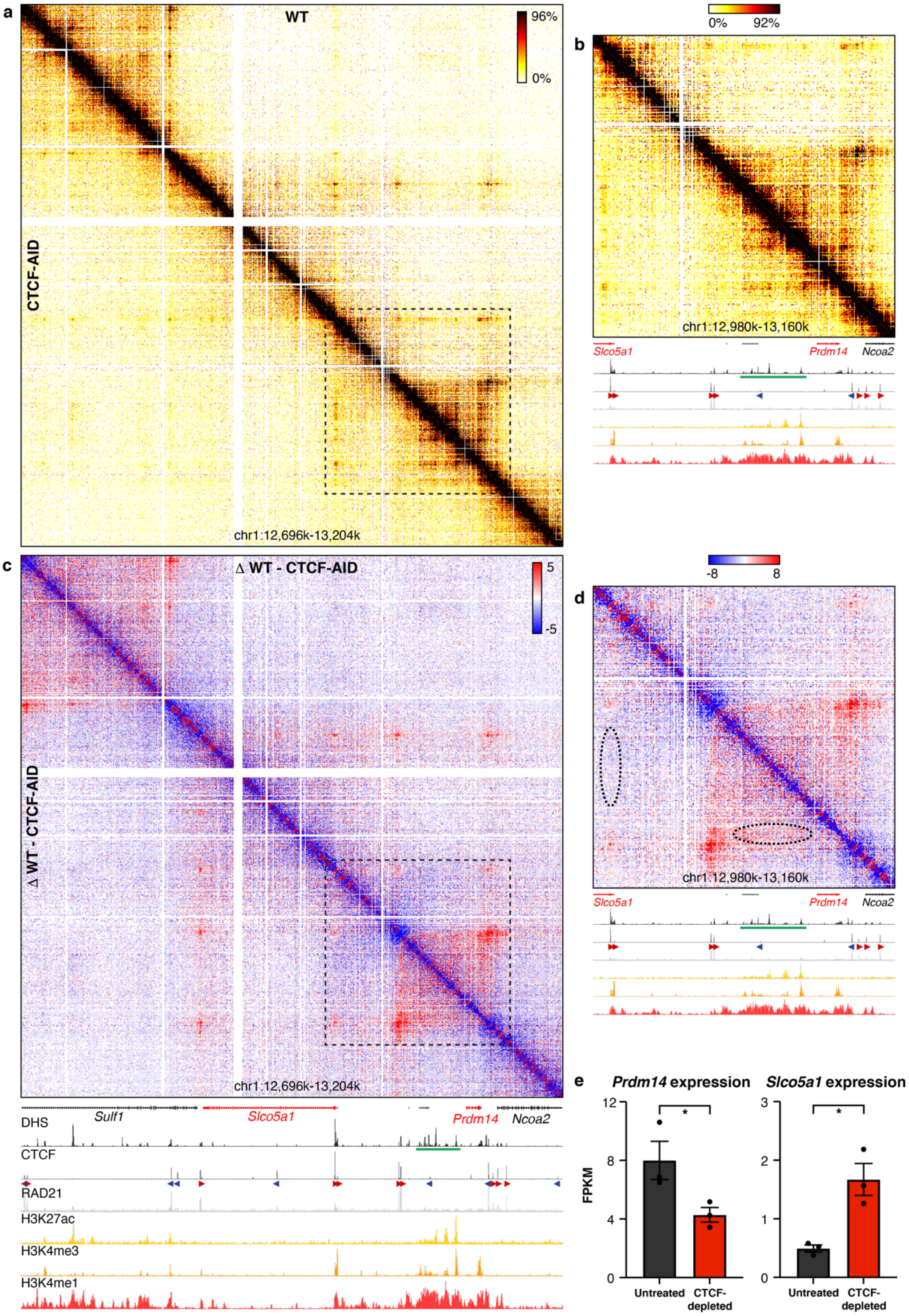

a. Tiled-MCC contact matrices of the *Prdm14* locus in wild type (WT) mES cells (top-right) and auxin-treated CTCF-AID mES cells (bottom-left) at 500 bp resolution. **b.** Zoomed view of the dashed region in panel a to highlight the interactions between the *Prdm14* and *Slco5a1* promoters and the super-enhancer. **c.** Differential contact matrix of the *Prdm14* locus in which interactions enriched in WT mES cells are indicated in red and interactions enriched in auxin-treated CTCF-AID mES cells are indicated in blue. **d.** Zoomed view of the dashed region in panel c to highlight the interactions between the *Prdm14* and *Slco5a1* promoters and the super-enhancer. **a-d.** Gene annotation (genes of interest in red, coding genes in black, non-coding genes in grey), DNase hypersensitive sites (DHS) and ChIP-seq data for CTCF, Cohesin (RAD21), H3K27ac, H3K4me3 and H3K4me1 are shown below the matrices. The axes of the DHS and ChIP-seq profiles below the contact matrices are scaled to signal and have the following ranges: DHS = 0-6.45; CTCF = 0-2167; RAD21 = 0-3032; H3K27ac = 0-44; H3K4me3 = 0-26; H3K4me1 = 0-2170. Enhancers of interest are indicated in green below the DHS profiles. The orientations of CTCF motifs at prominent CTCF-binding sites are indicated by arrowheads (forward orientation in red; reverse orientation in blue). The dashed ovals in panel d highlight interactions of interest, with the left oval indicating increased interactions between the *Slco5a1* promoter and the super-enhancer, and the right oval indicating decreased interactions between the *Prdm14* promoter and the super-enhancer, upon CTCF depletion. **e.** Expression of *Prdm14* and *Slco5a1* in untreated (left) and auxin-treated (right) CTCF-AID mES cells, derived from RNA-seq data, normalized for fragments per kilobase of transcript, per million mapped reads (FPKM). The bars represent the average of n=3 replicates and the error bars indicate the standard error of the mean. Significant (*) changes in expression are indicated. *Prdm14*: P = 0.0006; *Slco5a1*: P = 5.00E-05 (calculated using Cuffdiff analysis and adjusted for multiple comparisons, as previously decribed[10]). Source data are provided as a Source Data file.

Supplementary Figure 15. The effects of CTCF depletion on chromatin interactions and gene expression in the *Sox2* locus.

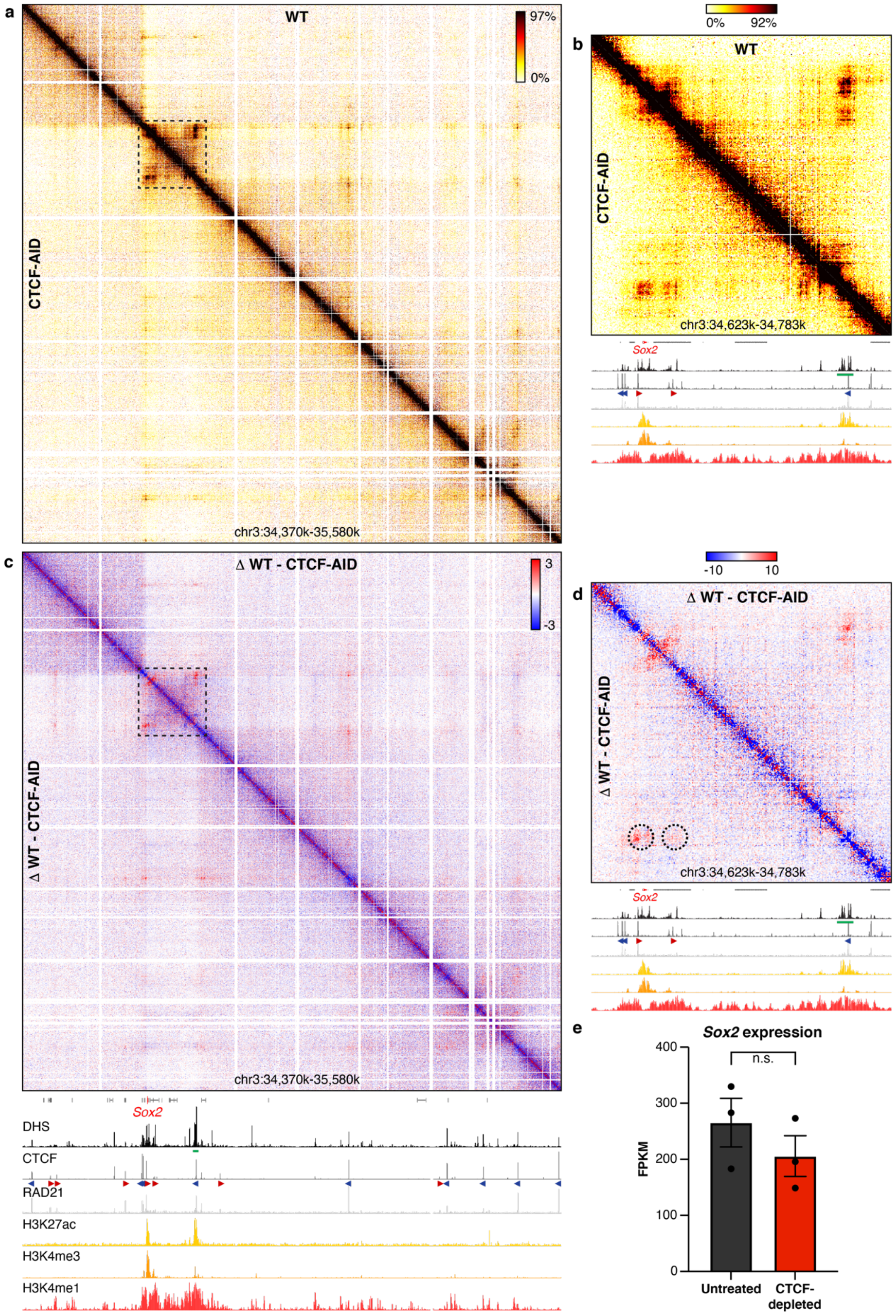

a. Tiled-MCC contact matrices of the *Sox2* locus in wild type (WT) mES cells (top-right) and auxin-treated CTCF-AID mES cells (bottom-left) at 500 bp resolution. **b.** Zoomed view of the dashed region in panel a to highlight the interactions between the *Sox2* promoter and its super-enhancer. **c.** Differential contact matrix of the *Sox2* locus in which interactions enriched in WT mES cells are indicated in red and interactions enriched in auxin-treated CTCF-AID mES cells are indicated in blue. **d.** Zoomed view of the dashed region in panel c to highlight the interactions between the *Sox2* promoter and its super-enhancer. **a-d.** Gene annotation (*Sox2* in red, coding genes in black, non-coding genes in grey), DNase hypersensitive sites (DHS) and ChIP-seq data for CTCF, Cohesin (RAD21), H3K27ac, H3K4me3 and H3K4me1 are shown below the matrices. The axes of the DHS and ChIP-seq profiles below panels a and c are scaled to signal and have the following ranges: DHS = 0-4.46; CTCF = 0-1833; RAD21 = 0-3318; H3K27ac = 0-48; H3K4me3 = 0-82; H3K4me1 = 0-1826. The axes of the DHS and ChIP-seq profiles below panel b and d have the same ranges as in panel a and c, except for the CTCF ChIP-seq profile, which is scaled 0-300. Enhancers of interest are indicated in green below the DHS profiles. The orientations of CTCF motifs at prominent CTCF-binding sites are indicated by arrowheads (forward orientation in red; reverse orientation in blue). Interactions of interest are highlighted with dashed circles in panel d, with the left circle indicating the interactions between the *Sox2* promoter and its super-enhancer, and the right circle indicating the interactions between CTCF-binding sites downstream of the promoter and the super-enhancer. Note that there is also a CTCF-binding site located directly upstream of the *Sox2* promoter and within the super-enhancer. The interactions between these elements are decreased upon CTCF depletion, but the reduction is less severe compared to the effects of depletion of cohesin. **e.** Expression of *Sox2* in untreated (left) and auxin-treated (right) CTCF-AID mES cells, derived from RNA-seq data, normalized for fragments per kilobase of transcript, per million mapped reads (FPKM). The bars represent the average of n=3 replicates and the error bars indicate the standard error of the mean. Non-significant (n.s.) changes in expression are indicated. P=0.098 (calculated using Cuffdiff analysis and adjusted for multiple comparisons, as previously decribed[10]). Source data are provided as a Source Data file.

Supplementary Figure 16. CTCF depletion does not have a strong effect on enhancer-promoter interactions and gene expression in the *Pou5f1* locus.

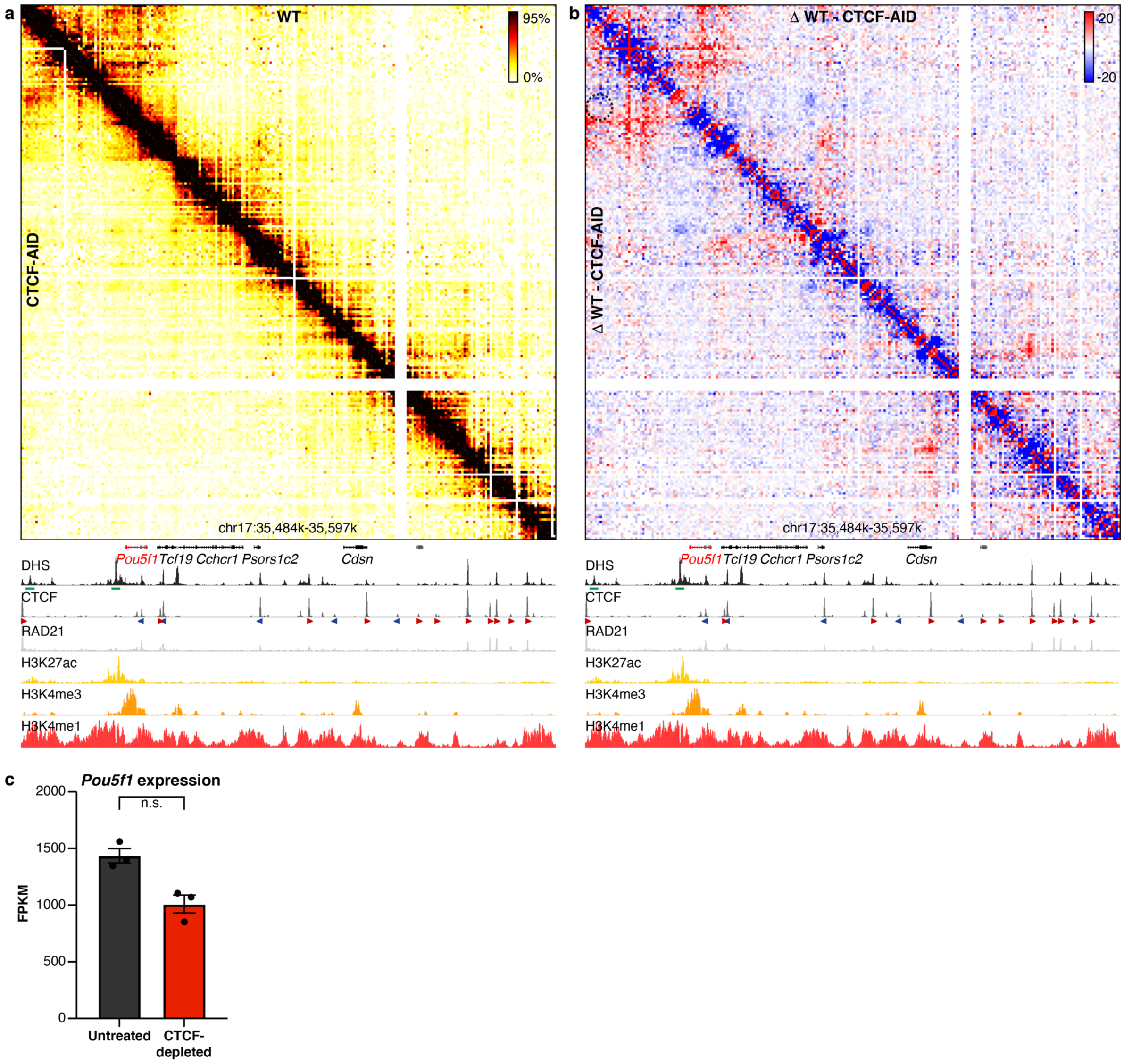

a. Tiled-MCC contact matrices of the *Pou5f1* locus in wild type mES cells (top-right) and auxin-treated CTCF-AID mES cells (bottom-left) at 500 bp resolution. **b.** Differential contact matrix of the *Pou5f1* locus in which interactions enriched in WT mES cells are indicated in red and interactions enriched in auxin-treated CTCF-AID mES cells are indicated in blue. **a-b.** Gene annotation (genes of interest in red, coding genes in black, non-coding genes in grey), DNase hypersensitive sites (DHS) and ChIP-seq data for CTCF, Cohesin (RAD21), H3K27ac, H3K4me3 and H3K4me1 are shown below the matrices. The axes of the DHS and ChIP-seq profiles are scaled to signal and have the following ranges: DHS = 0-8.65; CTCF = 0-2349; RAD21 = 0-4107; H3K27ac = 0-81; H3K4me3 = 0-64; H3K4me1 = 0-1641. Enhancers of interest are indicated in green below the DHS profiles. The orientations of CTCF motifs at prominent CTCF-binding sites are indicated by arrowheads (forward orientation in red; reverse orientation in blue). The dashed circle indicates the interactions between the *Pou5f1* promoter and the far upstream enhancer, which do not appear to change upon CTCF depletion. **c.** Expression of *Pou5f1* in untreated (left) and auxin-treated (right) CTCF-AID mES cells, derived from RNA-seq data, normalized for fragments per kilobase of transcript, per million mapped reads (FPKM). The bars represent the average of n=3 replicates and the error bars indicate the standard error of the mean. Non-significant (n.s.) changes in expression are indicated. P = 0.02515 (calculated using Cuffdiff analysis and adjusted for multiple comparisons, as previously decribed[10]). Source data are provided as a Source Data file.

Supplementary Figure 17. Micro-topologies of *cis*-regulatory elements in the *Prdm14* locus and their variable dependence on cohesin and CTCF.

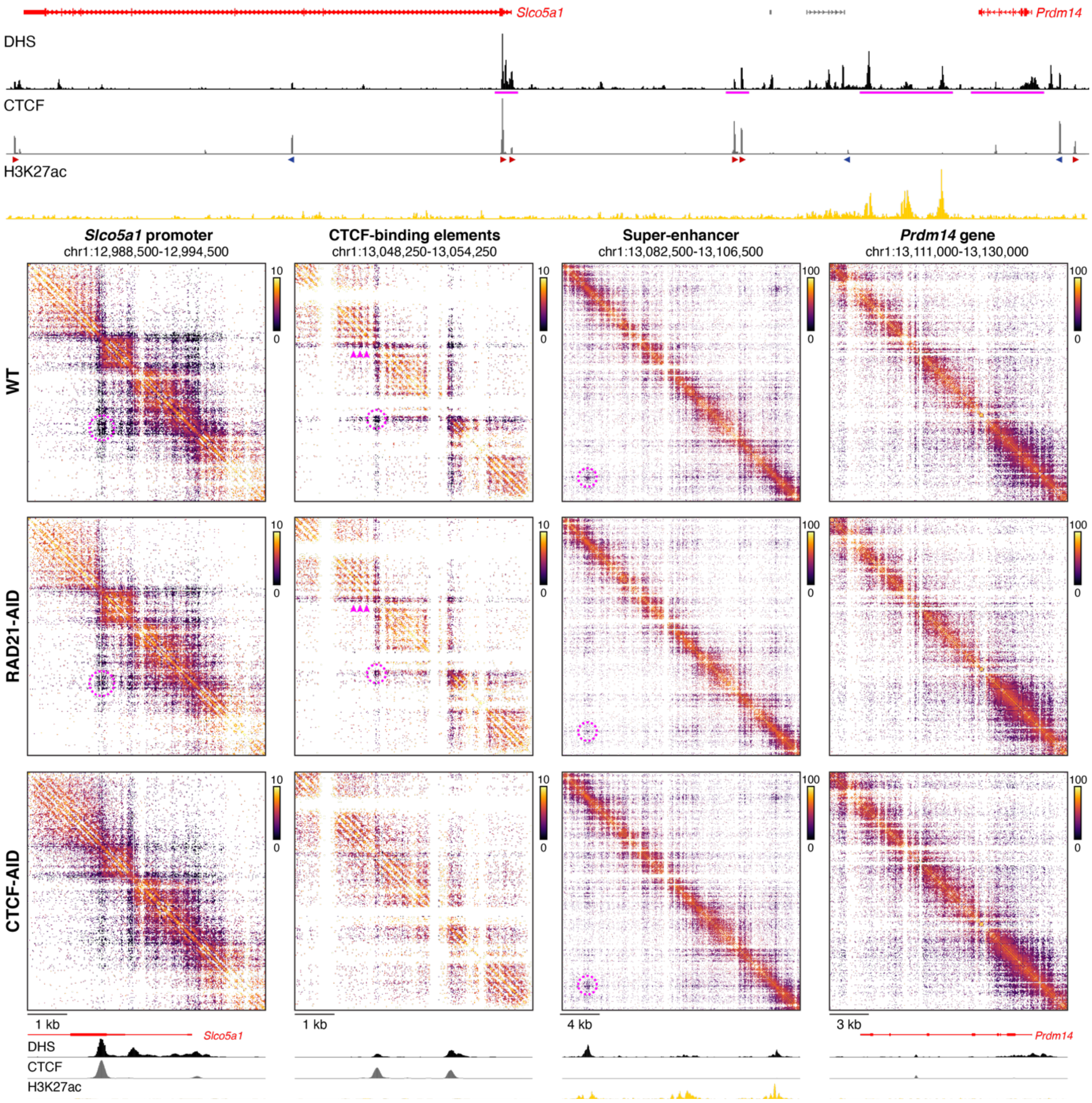

The contact matrices show ligation junctions identified by Tiled-MCC in the *Prdm14* locus at 20 bp resolution in wild-type (WT), auxin-treated RAD21-AID, and auxin-treated CTCF-AID mES cells. Gene annotation (genes of interest in red, coding genes in black, non-coding genes in grey), DNase hypersensitive sites (DHS), and ChIP-seq data for CTCF and H3K27ac for the extended *Prdm14* locus are shown above the matrices. The regions covered in the contact matrices are highlighted with magenta bars below the top DHS profile, and show a gene promoter, two CTCF-binding elements, a super-enhancer, and a gene. The axes of the top and bottom DHS and ChIP-seq profiles for CTCF and H3K27ac are fixed and have the following ranges: DHS = 0-6.5; CTCF = 0-2200; H3K27ac = 0-50. The orientations of CTCF motifs at prominent CTCF-binding sites are indicated by arrowheads (forward orientation in red; reverse orientation in blue). The magenta highlights in the contact matrices covering the *Slco5a1* promoter indicate enriched interactions between the promoter and a CTCF-binding site; the highlights in the contact matrices covering the CTCF-binding elements indicate phased nucleosomes and enriched interactions between the CTCF-binding elements; the highlights in the contact matrices covering the super-enhancer show enriched interactions between DHSs.

Supplementary Figure 18. Micro-topologies of CTCF-binding elements in the *Sox2* locus upon cohesin and CTCF depletion.

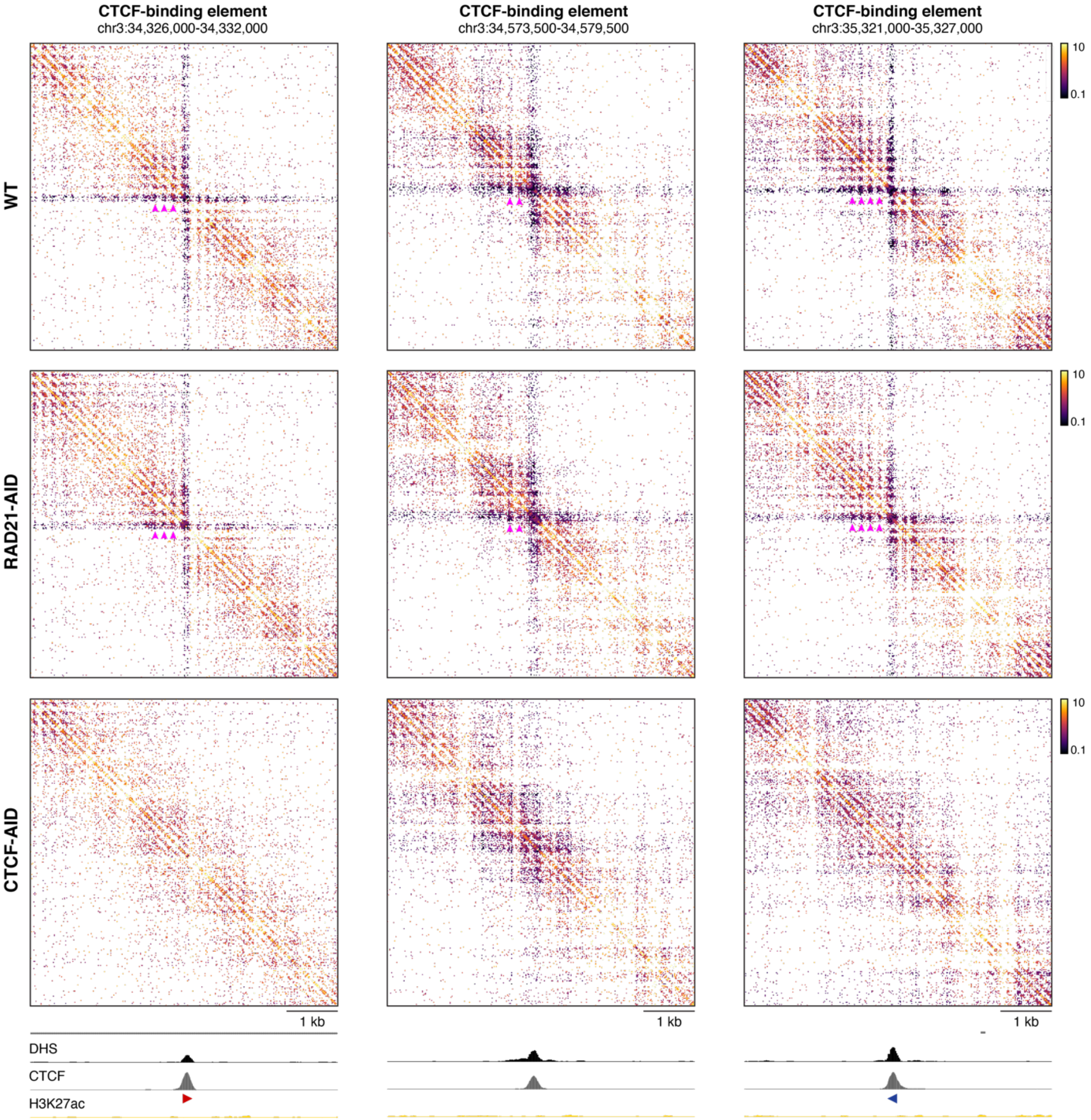

The contact matrices show ligation junctions identified by Tiled-MCC at CTCF-binding elements in the *Sox2* locus at 20 bp resolution in wild-type (WT), auxin-treated RAD21-AID, and auxin-treated CTCF-AID mES cells. Gene annotation (non-coding genes in grey), DNase hypersensitive sites (DHS), and ChIP-seq data for CTCF and H3K27ac are shown below the matrices. The axes of the DHS and ChIP-seq profiles for CTCF and H3K27ac are fixed and have the following ranges: DHS = 0-5; CTCF = 0-1500; H3K27ac = 0-50. The orientations of CTCF motifs with clear directionality are indicated by arrowheads (forward orientation in red; reverse orientation in blue). The magenta highlights indicate phased nucleosomes.

Supplementary Table 1. Comparison of sequencing and data depth in Tiled-MCC, Micro-C and Tiled-C experiments in the *Sox2* locus.

	Tiled-MCC	Micro-C	Tiled-C
**Total number of reads**	1,214,195,069	6,778,065,733	124,372,676
**Valid interactions per bp in *Sox2* region**	5.26	0.75	3.66
**Number of samples**	9	38	4

Overview of the sequencing and data depth of Tiled-MCC, Micro-C[1], and Tiled-C[4] experiments in the *Sox2* locus in wildtype mES cells. bp = base pair.

Supplementary Table 2. Comparison of Tiled-MCC and Micro-C data depth.

		Tiled-MCC		Micro-C	
**ROI**	**Size (bp)**	**Valid junctions in ROI**	**Valid junctions per bp**	**Valid junctions in ROI**	**Valid junctions per bp**
Sox2	1,280,000	6,730,085	5.26	961,509	0.75
Prdm14	510,000	3,860,315	7.57	606,305	1.19
Nanog	250,000	1,740,491	6.96	336,743	1.35
Pou5f1	115,000	1,071,663	9.32	249,343	2.17

Overview of the depth of Tiled-MCC and Micro-C[1] data in wild type mES cells. ROI = region of interest; bp = base pair.

References

1. Hsieh, T.-H.S. *et al.* Resolving the 3D Landscape of Transcription-Linked Mammalian Chromatin Folding. *Molecular Cell* 78, 339-553 (2020).
2. Krietenstein, N. *et al.* Ultrastructural Details of Mammalian Chromosome Architecture. *Molecular Cell* 78, 554-565 (2020).
3. Hua, P. *et al.* Defining genome architecture at base-pair resolution. *Nature* 595, 125-129 (2021).
4. Oudelaar, A.M. *et al.* Dynamics of the 4D genome during in vivo lineage specification and differentiation. *Nature Communications* 11, 2722 (2020).
5. Rhodes, J.D.P. *et al.* Cohesin Disrupts Polycomb-Dependent Chromosome Interactions in Embryonic Stem Cells. *Cell Reports* 30, 820-835 (2020).
6. Vos, E.S.M. *et al.* Interplay between CTCF boundaries and a super enhancer controls cohesin extrusion trajectories and gene expression. *Molecular Cell* 81, 3082-3095 (2021).
7. Hnisz, D. *et al.* Convergence of Developmental and Oncogenic Signaling Pathways at Transcriptional Super-Enhancers. *Molecular Cell* 58, 362-370 (2015).
8. Bleckwehl, T. *et al.* Enhancer-associated H3K4 methylation safeguards in vitro germline competence. *Nature Communications* 12, 5771 (2021).
9. Blinka, S., Reimer, Michael H., Pulakanti, K. & Rao, S. Super-Enhancers at the Nanog Locus Differentially Regulate Neighboring Pluripotency-Associated Genes. *Cell Reports* 17, 19-28 (2016).
10. Nora, E.P. *et al.* Targeted Degradation of CTCF Decouples Local Insulation of Chromosome Domains from Genomic Compartmentalization. *Cell* 169, 930-944 (2017).
