# Supplementary material for: Analysis of sub-kilobase chromatin topology reveals nano-scale regulatory interactions with variable dependence on cohesin and CTCF
Source: Nat Commun. 2022 Apr 19;13:2139. doi: 10.1038/s41467-022-29696-5 (PMC9019034; doi:10.1038/s41467-022-29696-5)
Supplement: Supplementary file 3 — Reporting Summary [file 41467_2022_29696_MOESM3_ESM.pdf]

nature portfolio

Corresponding author(s): A. Marieke Oudelaar

Last updated by author(s): Mar 12, 2022

Reporting Summary

Nature Portfolio wishes to improve the reproducibility of the work that we publish. This form provides structure for consistency and transparency in reporting. For further information on Nature Portfolio policies, see our Editorial Policies and the Editorial Policy Checklist.

Statistics

For all statistical analyses, confirm that the following items are present in the figure legend, table legend, main text, or Methods section.

n/a	Confirmed	
[ ]	[x]	The exact sample size (*n*) for each experimental group/condition, given as a discrete number and unit of measurement
[ ]	[x]	A statement on whether measurements were taken from distinct samples or whether the same sample was measured repeatedly
[ ]	[x]	The statistical test(s) used AND whether they are one- or two-sided<br>*Only common tests should be described solely by name; describe more complex techniques in the Methods section.*
[x]	[ ]	A description of all covariates tested
[x]	[ ]	A description of any assumptions or corrections, such as tests of normality and adjustment for multiple comparisons
[ ]	[x]	A full description of the statistical parameters including central tendency (e.g. means) or other basic estimates (e.g. regression coefficient) AND variation (e.g. standard deviation) or associated estimates of uncertainty (e.g. confidence intervals)
[ ]	[x]	For null hypothesis testing, the test statistic (e.g. *F*, *t*, *r*) with confidence intervals, effect sizes, degrees of freedom and *P* value noted<br>*Give P values as exact values whenever suitable.*
[x]	[ ]	For Bayesian analysis, information on the choice of priors and Markov chain Monte Carlo settings
[x]	[ ]	For hierarchical and complex designs, identification of the appropriate level for tests and full reporting of outcomes
[x]	[ ]	Estimates of effect sizes (e.g. Cohen's *d*, Pearson's *r*), indicating how they were calculated

Our web collection on statistics for biologists contains articles on many of the points above.

Software and code

Policy information about availability of computer code

Data collection	Illumina NovaSeq and NextSeq 550.
Data analysis	MCC pipeline (https://github.com/jojdavies/Micro-Capture-C), based on scripts available for academic use through the Oxford University Innovation software store (https://process.innovation.ox.ac.uk/software/p/16529a/micro- capture-c-academic/1); Trim Galore v.0.3.1; FLASH v.1.2.11; BLAT v.35; Bowtie2 v.2.3.5; HiC-Pro v.2.11.1; oligo design tool v.0.1.1b (https://oligo.readthedocs.io/en/latest/).

For manuscripts utilizing custom algorithms or software that are central to the research but not yet described in published literature, software must be made available to editors and reviewers. We strongly encourage code deposition in a community repository (e.g. GitHub). See the Nature Portfolio guidelines for submitting code & software for further information.

Data

Policy information about availability of data

All manuscripts must include a data availability statement. This statement should provide the following information, where applicable:

- Accession codes, unique identifiers, or web links for publicly available datasets
- A description of any restrictions on data availability
- For clinical datasets or third party data, please ensure that the statement adheres to our policy

The Tiled-MCC data generated in this study have been deposited in the NCBI Gene Expression Omnibus under accession code GSE181694 [https://www.ncbi.nlm.nih.gov/geo/query/acc.cgi?acc=GSE181694]. The RNA-seq data in RAD21-AID cells used in this study are available in the ArrayExpress Archive under accession code E-MTAB-7818 [https://www.ebi.ac.uk/arrayexpress/experiments/E-MTAB-7818/]. The RNA-seq data in CTCF-AID cells used in this study are available in the NCBI Gene Expression Omnibus under accession code GSE98671 [https://www.ncbi.nlm.nih.gov/geo/query/acc.cgi?acc=GSE98671]. The ChIP-seq data for CTCF in mES cells used in this study are available in the NCBI Gene Expression Omnibus under accession code GSE30203 [https://www.ncbi.nlm.nih.gov/geo/query/

acc.cgi?acc=GSE30203]. The ChIP-seq data for RAD21 in mES cells used in this study are available in the NCBI Gene Expression Omnibus under accession code GSE94452 [https://www.ncbi.nlm.nih.gov/geo/query/acc.cgi?acc=GSE94452]. The ChIP-seq data for H3K4me1 in mES cells used in this study are available in the NCBI Gene Expression Omnibus under accession code GSE27844 [https://www.ncbi.nlm.nih.gov/geo/query/acc.cgi?acc=GSE27844]. ChIP-seq data for H3K27ac and H3K4me3 and DNase I hypersensitivity data in mES cells were accessed via ENCODE.

Field-specific reporting

Please select the one below that is the best fit for your research. If you are not sure, read the appropriate sections before making your selection.

☒ Life sciences ☐ Behavioural & social sciences ☐ Ecological, evolutionary & environmental sciences

For a reference copy of the document with all sections, see nature.com/documents/nr-reporting-summary-flat.pdf

Life sciences study design

All studies must disclose on these points even when the disclosure is negative.

Sample size	The Tiled-MCC data presented in the manuscript represent the averages of 9 replicates for WT samples, 6 replicates for auxin-treated RAD21-AID samples, and 6 replicates for auxin-treated CTCF-AID samples. These sample sizes were chosen to generate data at sufficient depth and assess differences between conditions robustly. These sample sizes are sufficient, since the observed biological effects of interest are clearly detectable between conditions and robust across replicates (Supplementary Figures 1, 10, 13).
Data exclusions	No data were excluded.
Replication	Tiled-MCC experiments were performed in multiple biological replicates as described and all attempts were successful. Immunoblots were performed independently three times with similar results.
Randomization	Samples were randomly allocated into different experimental groups prior to their treatment with auxin.
Blinding	All samples were analyzed with the same pipeline, in which interactions are detected by scripts without interference of the researchers. Since potential expectations of the researchers cannot influence the data analysis and results, blinding is not relevant to this study.

Reporting for specific materials, systems and methods

We require information from authors about some types of materials, experimental systems and methods used in many studies. Here, indicate whether each material, system or method listed is relevant to your study. If you are not sure if a list item applies to your research, read the appropriate section before selecting a response.

Materials & experimental systems

n/a	Involved in the study	
☐	☒	Antibodies
☐	☒	Eukaryotic cell lines
☒	☐	Palaeontology and archaeology
☒	☐	Animals and other organisms
☒	☐	Human research participants
☒	☐	Clinical data
☒	☐	Dual use research of concern

Methods

n/a	Involved in the study	
☒	☐	ChIP-seq
☒	☐	Flow cytometry
☒	☐	MRI-based neuroimaging

Antibodies

Antibodies used	Anti-RAD21 antibody (Abcam, ab154769), anti-CTCF antibody (Abcam, ab70303), anti-histone H3 antibody (HRP) (Abcam, ab21054), Goat Anti-Rabbit IgG H&L (HRP) (Abcam, ab205718).
Validation	Validation was performed by the manufacturer (Abcam). The antibodies were purified using immunogen affinity and validated by immunoprecipitation, immunohistochemical analysis, and western blotting.

Eukaryotic cell lines

Policy information about cell lines

Cell line source(s)	Mouse embryonic stem (mES) ES-E14TG2a.4 cells (gift from Doug Higgs), mES-RAD21-mAID-eGFP cells (Rhodes et al. Cell Reports 2021), mES-CTCF-AID-eGFP cells (Nora et al. Cell 2017).

Authentication	ES-E14TG2a.4 cells are used for the generation of genetically modified mouse lines.
Mycoplasma contamination	All cell lines tested negative for mycoplasma contamination.
Commonly misidentified lines (See ICLAC register)	No commonly misidentified lines were used.
